# *Lycium barbarum*-probiotic synergy alleviates chemotherapy-induced cancer-related fatigue via gut microbiota-metabolic axis regulation in mice

**DOI:** 10.3389/fnut.2025.1613132

**Published:** 2025-07-02

**Authors:** Hexiang Feng, Linjun Zhong, Xinjian Yang, Haokun Wu, Qiankai Sun, Hao Wei, Yinbo He, Linsen Zhou, Yusong Zhang, Mei Li, Yu Sun

**Affiliations:** ^1^West China School of Medicine, Sichuan University, Chengdu, China; ^2^Department of Thoracic Oncology, West China Hospital, Sichuan University, Chengdu, China; ^3^Department of Radiotherapy, Cancer Center, West China Hospital, Sichuan University, Chengdu, China; ^4^Department of Medical Oncology, Cancer Center, West China Hospital, Sichuan University, Chengdu, China

**Keywords:** probiotics, *Lycium barbarum*, gut microbiota, chemotherapy, cancer-related fatigue

## Abstract

**Introduction:**

This study investigated the effects of a compound preparation combining *Lycium barbarum* and probiotics (LB-Pro) on chemotherapy-induced cancer-related fatigue (CRF) in a murine model. The aim was to explore potential mechanisms related to the gut microbiota-metabolic axis.

**Materials and methods:**

A CRF model was established in C57BL/6NCr mice using 5-fluorouracil. Mice were divided into four groups receiving varying concentrations of LB-Pro. Over 14 days, interventions were administered, followed by treadmill exhaustion tests to assess fatigue levels. Body weight, serum biomarkers (TNF-*α*, GSH-Px, NAD-MDH, SOD), and gut microbiota composition were analyzed to evaluate physiological and metabolic changes.

**Results:**

Administration of medium- and high-concentration LB-Pro significantly improved fatigue-related outcomes, including prolonged exhaustion times and enhanced antioxidant enzyme activities, compared to the control group. Low-concentration LB-Pro showed limited efficacy. Gut microbiota analysis revealed alterations in microbial composition, including enrichment of short-chain fatty acid-producing taxa, and metabolic pathways associated with energy metabolism and antioxidant defense were upregulated in probiotic-treated groups.

**Conclusion:**

LB-Pro alleviated chemotherapy-induced CRF in mice, likely through modulation of gut microbiota and enhancement of mitochondrial energy metabolism and antioxidant systems. These findings highlight the potential of integrative approaches combining traditional Chinese medicine and probiotics for managing CRF, emphasizing the gut microbiota-metabolic axis as a key therapeutic target.

## 1 Introduction

Cancer-related fatigue (CRF) is a prevalent and persistent condition in cancer patients, representing one of the most common adverse reactions during diagnosis and treatment (1). Characterized by weakness, depressed mood, cognitive decline, loss of interest, inability to perform previously manageable tasks, and fatigue that cannot be alleviated by adequate sleep or rest, CRF severely impacts patients’ nutritional status and functional capacity (2). Epidemiological surveys indicate CRF incidence rates of 70–100% in cancer patients, including 80–96% of chemotherapy recipients and 60–90% of radiotherapy patients (3, 4), which significantly damages their quality of life. What’s more, CRF can occur before treatment and persist throughout the entire treatment process, and symptoms may even endure for years after treatment completion. The pathological mechanisms of CRF are complex, involving multiple systems such as immune, neural, and endocrine systems (5, 6). Given its clinical characteristics, no universally accepted optimal treatment exists. Current clinical approaches include symptomatic management through immune regulation, antidepressants, nutritional support, sleep improvement, and exercise therapy (1, 7, 8).

The gut microbiota plays a critical role in numerous physiological functions, particularly metabolism, inflammation, and immunity. Emerging evidence suggests potential links between CRF severity and gut microbiota composition in cancer patients. For example, Wei et al. (9) reported that advanced lung cancer patients receiving first-line chemotherapy with severe CRF exhibited increased pro-inflammatory gut microbial taxa, while those with mild CRF showed elevated anti-inflammatory taxa. These findings suggest that CRF may be ameliorated through gut microbiota modulation. Meanwhile, traditional Chinese medicine has demonstrated unique advantages and efficacy in CRF treatment. *Lycium barbarum* contain multiple bioactive components, including *Lycium barbarum* polysaccharides (LBP), carotenoids, flavonoids, and alkaloids, and among these, LBP is recognized as the core active constituent (10, 11). Studies have demonstrated that *Lycium barbarum* alleviates CRF through diverse mechanisms, such as suppression of inflammatory cascades (12), enhancement of mitochondrial function (13), neuroprotection, and modulation of the hypothalamic–pituitary–adrenal (HPA) axis (14). Additionally, due to the presence of dietary fiber, polyphenols, and polysaccharides, *Lycium barbarum* exhibits prebiotic-like properties by modulating the abundance, distribution, and metabolic profiles of gut microbiota. This modulation promotes the activity of beneficial microbes (15, 16).

This study used a compound preparation of *Lycium barbarum* and probiotics (hereafter “LB-Pro”), and established a CRF model undergoing chemotherapy in C57BL/6NCr mice, investigating the effects of different concentrations of the LB-Pros on fatigue levels, physiological functions, and gut microbiota in C57BL/6NCr mice.

## 2 Materials and methods

### 2.1 Preparation of experimental animals

In this experiment, we selected 8-week-old male C57BL/6NCr mice because this strain exhibits high genomic homology and metabolic functions that closely resemble those of humans. Furthermore, the C57BL/6NCr strain demonstrates elevated complement activity and significant interferon production, enabling the simulation of human immune microenvironments and inflammatory responses during tumor induction. This makes it particularly suitable for investigating the immunometabolic mechanisms underlying CRF. Additionally, C57BL/6 mice show high sensitivity to chemically induced models, and their low spontaneous tumor incidence allows for synchronized study of tumor burden and fatigue symptoms through regulated carcinogen exposure. Moreover, the glucose metabolism of C57BL/6NCr mice is closer to that of humans, with elevated plasma urea and electrolyte levels, which can simulate metabolic disturbances commonly observed in CRF patients, such as lactate accumulation and energy depletion.

The mice were maintained in a specific-pathogen-free (SPF) environment, housed in groups of five per cage with aspen wood shavings as bedding material and aspen wooden blocks provided for gnawing. Environmental conditions were strictly controlled, with a temperature range of 22–26°C, humidity at 40–70%, and a 12-h light/dark cycle. The mice were fed a nutritionally complete maintenance diet (provided by Beijing Keaoxieli Feed Co., Ltd. Beijing, China) and had ad libitum access to sterilized drinking water. For drug administration, the target compound was mixed into the drinking water at a calculated dose based on the daily water consumption of the mice, with precise volume adjustments to ensure consistent daily intake. Prior to the experiment, all mice underwent a 5–7-day acclimatization period to adapt to the laboratory environment.

A total of 24 male C57BL/6NCr mice were used in this study. The mice were randomly divided into four groups according to the concentration of LB-Pro: Chemotherapy-Control group (CC group), Chemotherapy-Low concentration probiotic group (CL group), Chemotherapy-Medium concentration probiotic group (CM group), and Chemotherapy-High concentration probiotic group (CH group).

### 2.2 Preparation of LB-Pro

The probiotic sample contained active probiotics at a concentration of 10 billion CFU per strip. Ingredients included *Lycium barbarum* powder, fructooligosaccharides, sea cucumber oligopeptide powder, yeast *β*-glucan, *Lactobacillus paracasei LPC-37*, and *Bifidobacterium animalis subsp. lactis Bi-07*.

The formulation integrates prebiotics, probiotics, and bioactive peptides to create a multifaceted approach to health promotion. *Lycium barbarum* powder serves as a potent antioxidant and anti-inflammatory agent, modulating gut microbiota composition by promoting beneficial taxa (17). As a prebiotic, fructooligosaccharides act as a fermentable substrate, selectively stimulating the growth of *Lactobacillus* and *Bifidobacterium* strains in the formulation, thereby enhancing their probiotic efficacy (18). Sea cucumber oligopeptide powder exhibits immunomodulatory and anti-fatigue properties, improving exercise capacity by facilitating muscle recovery and reducing lactate accumulation (19). Yeast *β*-glucan, a well-characterized immunomodulatory compound, activates innate immune cells (e.g., macrophages, neutrophils) via dectin-1 receptors, enhancing phagocytosis and cytokine production (20). *Lactobacillus paracasei LPC-37* is known for its ability to adhere to intestinal epithelial cells, produce antimicrobial substances (e.g., bacteriocins), and modulate immune responses. *Bifidobacterium animalis subsp. lactis Bi-07*, a widely studied probiotic, which can produce anti-inflammatory metabolites and regulate immune cell function (21).

For the CC group, 20 mL of distilled water was administered as a placebo. For the CL, CM, and CH groups, LB-Pro samples were prepared at doses of 21.5 mg/kg, 100 mg/kg, and 500 mg/kg body weight, respectively. The samples were dissolved in 20 mL of distilled water, thoroughly mixed. Then LB-Pro was added to the drinking water of mice for them to drink freely, without gastric perfusion.

### 2.3 Animal experiments

To establish a CRF model undergoing chemotherapy in mice, 5-fluorouracil (30 mg/kg) was intraperitoneally injected once daily for 5 consecutive days before probiotic intervention. And then this treatment induced fatigue manifestations in mice such as reduced spontaneous activity in the open field and decreased the running ability. After 5 days of simulated chemotherapy, mice received either placebo or LB-Pro twice daily (morning and evening) for 14 days.

Following the 14-day intervention period, these mice underwent 3 days of exercise adaptation training on a 5° inclined treadmill. The training protocol involved running at speeds of 10, 15, 20, 24, and 28 m/min for 10 min each. On the 4th day, an exhaustion test was conducted. The mice ran on the treadmill at a speed of 28 m/min and started to time. When the mice were tired, they were stimulated with the tail of the needle, each time for 1 ~ 2 s, and the stimulation frequency was more than 4 times per second. Exhaustion was confirmed if the mice remained in the posterior one-third of the treadmill 3 times or more and failed to respond to 10 s of continuous stimulation. The total running time until exhaustion was recorded.

### 2.4 Data collection

During the 14-day intervention period, mouse body weights were measured before morning feeding every 2–3 days. Weight data from day 1, day 8, and day 15 were used to represent baseline, week 1, and week 2 weights, respectively. Fresh fecal samples were collected on days 1, 7, and 14, immediately snap-frozen in liquid nitrogen, and stored at-80°C.

On the day of the exhaustion test (day 4 of exercise training), orbital blood samples were collected to measure serum TNF-*α* concentration, GSH-Px activity, NAD-MDH activity, and SOD activity. Additionally, blood samples were collected as serum using one-time-use yellow-tipped blood collection tubes. After blood collection, the samples were allowed to stand at room temperature for 30 min. Subsequently, the serum was separated using a cold centrifuge (4°C) at 3,500 rpm for 10 min. The supernatant above the clotting agent was carefully collected as the final serum sample. TNF-*α* levels were determined using an ELISA kit, while GSH-Px, NAD-MDH, and SOD activities were measured via colorimetric assay kits. The Mouse TNF-α ELISA Kit (Cat. No: E-EL-M3063), GSH-Px Activity Assay Kit (Cat. No: E-BC-K096-M), NAD-MDH Activity Assay Kit (Cat. No: E-BC-K561-M), and T-SOD Activity Assay Kit (WST-1 Method, Cat. No: E-BC-K020-M) were all sourced from Elabscience Biotechnology Co., Ltd. (Wuhan, China).

### 2.5 Statistical analysis

Data are presented as mean ± standard deviation (SD). Statistical analysis was performed using SPSS software (version 24.0) with a significance level set at *p* < 0.05. For comparisons of body weight, running time, and serum biochemical markers between groups, we used one-way ANOVA combined with post-hoc tests, where results of multiple comparisons were primarily based on LSD. For longitudinal analysis of body weight on day 1, week 1, and week 2, repeated measures ANOVA was applied. Regarding microbial analysis, this study employed 16S rDNA sequencing, rarefaction curve analysis, alpha- and beta-diversity analysis, LEfSe taxonomic differential analysis, and PICRUSt2 functional prediction integrated with random forest machine learning. Gut microbiota analysis was performed using 16S rDNA profiling technology by Rhonin Biosciences Co., Ltd. (Chengdu, China), which generated raw sequencing data and analysis results.

## 3 Results and discussion

### 3.1 Analysis of body weight changes

**Figure 1:**
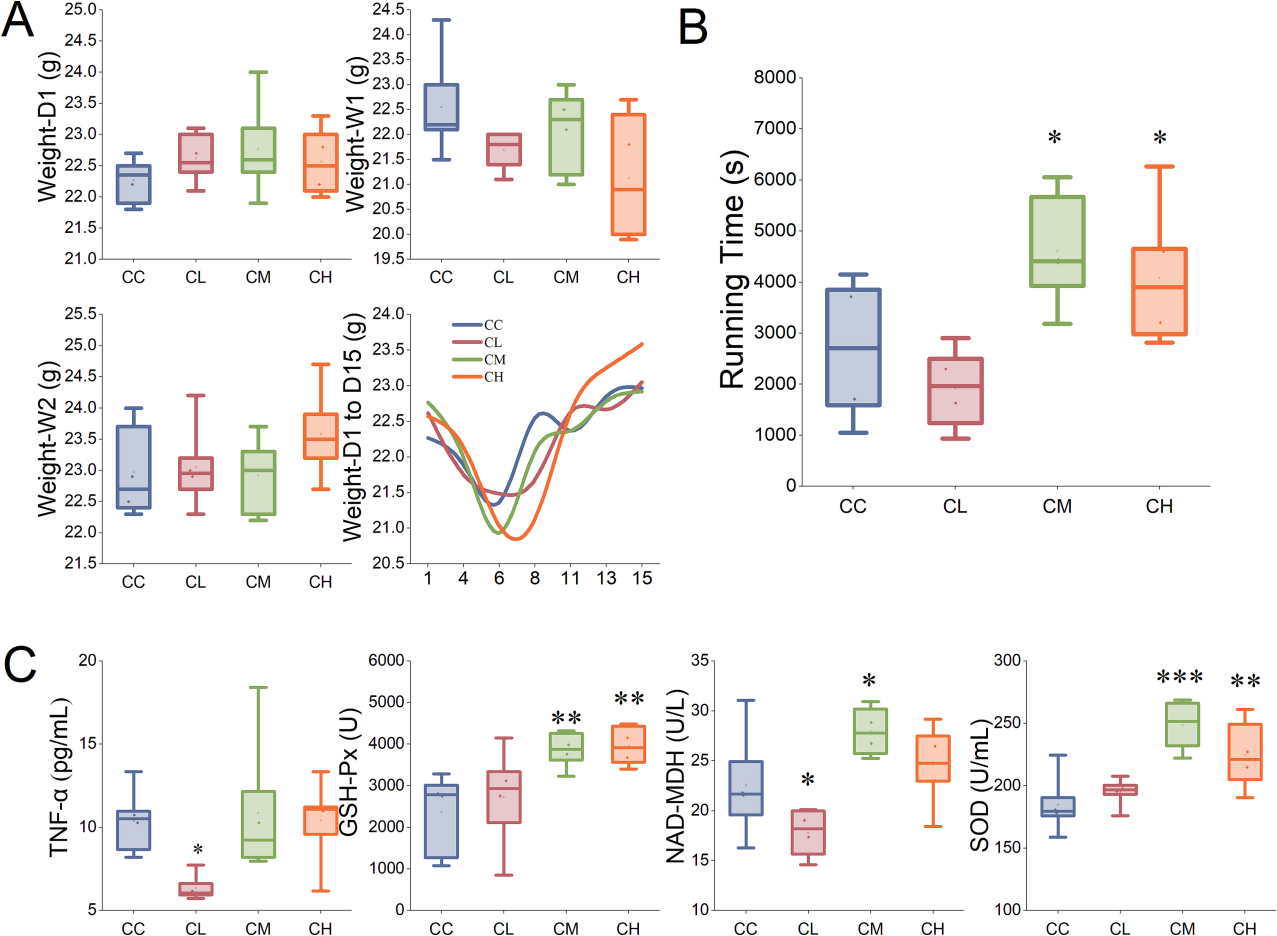
Comparison of body weight, running time and serum biochemical markers. **(A)** Body weights of mice on Day 1 (Weight-D1), Day 8 (Weight-W1), and Day 15 (Weight-W2), and the changes of body weight over 2 weeks. **(B)** Running time for mice during the exhaustion test. **(C)** Serum TNF-*α* content, GSH-Px activity, NAD-MDH activity, and SOD activity measured after the exhaustion test. CC, Chemotherapy-Control group; CL, Chemotherapy-Low concentration LB-Pro group; CM, Chemotherapy-Medium concentration LB-Pro group; CH, Chemotherapy-High concentration LB-Pro group; ^*^p < 0.05; ^**^p < 0.01; ^***^p < 0.001.

As shown in Figure 1A, there were no significant differences in the initial body weights of the four groups of mice (*p* > 0.05). During the first week of intragastric administration of probiotics/placebo, the body weights of the four groups of mice all showed a downward trend, but the differences between the groups were not statistically significant (*p* > 0.05). This may be because the injection of 5-fluorouracil caused an inflammatory stress response, leading to a disorder in the metabolic function of the mice and resulting in weight loss. And it is worth noting that in the preliminary experiment, when 60 mg/kg of 5-fluorouracil was used, all the mice died within 2 weeks, indicating that the toxicity of this dose of 5-fluorouracil was too high.

During the second week of intragastric administration of probiotics/placebo, the body weights of the four groups of mice all increased significantly and were higher than the initial body weights. This may be the result of the combined effects of the mice’s self-repair and probiotic intervention.

Then Repeated Measures ANOVA was performed on the body weights of the mice on the first day, at the end of the first week, and at the end of the second week. Mauchly’s Test of Sphericity indicated that the data did not meet the Sphericity Assumption (*W* = 0.710, *p* = 0.038). Therefore, the Greenhouse–Geisser correction was applied (*ε* = 0.775).

The within-subject effect test showed that the main effect of time was significant [*F*(1.55, 30.99) = 6.581, *p* < 0.001], and the interaction effect between time and concentration was also significant [*F*(4.65, 30.99) = 6.902, *p* < 0.001]. While the between-subject effect test showed that the main effect of concentration was not significant [*F*(3, 20) = 0.122, *p* = 0.946]. The test results indicated that the body weights of the mice changed significantly over time (from Day 1 to Day 15), while there was no statistically significant overall effect of probiotic concentration on body weight, therefore the effect of probiotics might be time-dependent. These results were basically consistent with the findings shown in Figure 1A and the experimental observations.

### 3.2 Analysis of running time

To evaluate the fatigue level of the mice, an incremental-load treadmill experiment was conducted on the mice, with continuous stimulation given during the experiment until the mice were exhausted. As shown in Figure 1B, compared with the CC group, the exhaustion times of the mice in the CM group and the CH group were significantly prolonged (*p* < 0.05), while the exhaustion time of the mice in the CL group did not improve significantly (*p* > 0.05).

This indicates that medium (100 mg/kg) and high (500 mg/kg) concentrations of LB-Pros can effectively relieve CRF caused by chemotherapy in C57BL/6NCr mice, and the medium concentration has the best effect among the three different concentrations.

According to the comparison of running times among the groups of mice, we can clearly conclude that the anti-CRF effect of LB-Pro is dose-dependent. Only when the dose of LB-Pro administered to the mice reaches a certain level can its anti-fatigue effect significantly surpass the damage caused by chemotherapeutic agents to the mice. Notably, according to the experimental results, the anti-fatigue effect of high-concentration LB-Pro is less pronounced than that of medium-dose LB-Pro, indicating potential toxicity at excessive doses.

**Figure 2:**
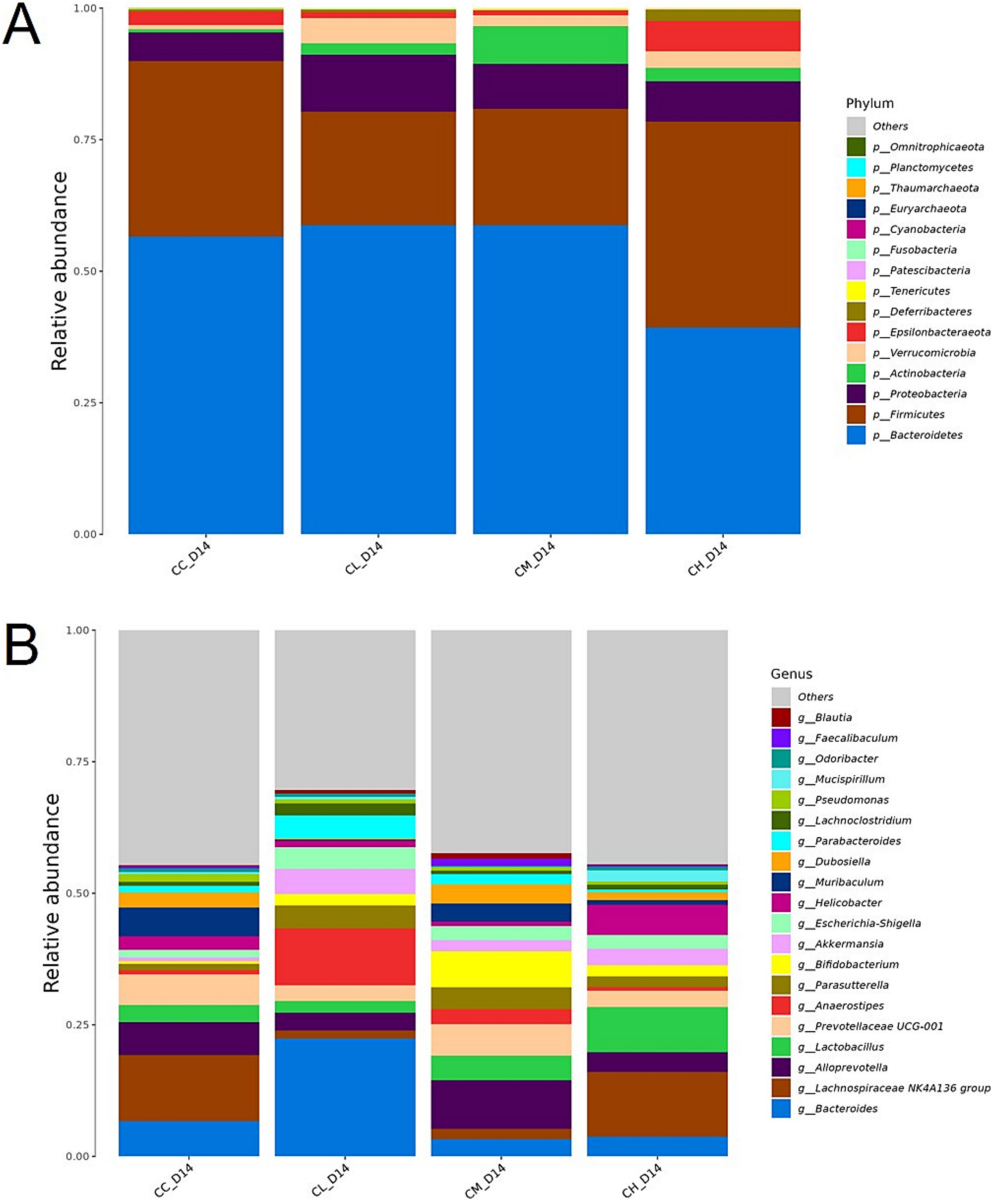
Relative abundance of gut microbiota on Day 14. **(A)** Phylum-level relative abundance. **(B)** Genus-level relative abundance. *p*_, phylum level; *g*_, genus level; CC, Chemotherapy-Control group; CL, Chemotherapy-Low concentration LB-Pro group; CM, Chemotherapy-Medium concentration LB-Pro group; CH, Chemotherapy-High concentration LB-Pro group.

Lou et al. (22) found in their study that in a murine model of liver injury, high concentrations of probiotics (e.g., *Lactobacillus Li01*) could disrupt gut microbiota balance, leading to a reduction in specific bacterial taxa (e.g., *Bacteroides*) and overproliferation of conditionally pathogenic bacteria (e.g., *Enterobacteriaceae*). This dysbiosis may exacerbate inflammation through the release of LPS and significantly increase gut barrier permeability. This partially aligns with our experimental results; as shown in Figure 2, the relative abundance of *p_Bacteroidetes* in the high-dose LB-Pro group (CH group) is notably lower than in other groups. Arribas et al. (23) reported in their study on an LPS-induced sepsis model that excessive administration of probiotics (e.g., *E. coli Nissle 1917*) might activate macrophages to produce excessive ROS, leading to oxidative stress. Tomasik et al. (24) further demonstrated that excessive probiotic administration could interfere with host metabolism. For example, overcolonization by *Bifidobacterium* species might suppress the growth of butyrate-producing bacteria, impair glucose metabolism, and worsen insulin resistance. Additionally, the accumulation of fermentation byproducts (e.g., D-lactic acid) from high-concentration probiotics could result in metabolic acidosis. These findings provide insights and guidance for investigating the potential toxicity of high-dose LB-Pro. In subsequent experiments, we can analyze the dose-dependent mechanisms and toxicity of LB-Pro by measuring plasma LPS-binding protein levels, ROS levels, and serum lactate concentrations.

Additionally, in terms of experimental design, the fatigue assessment was limited to physical performance (running time), neglecting the multidimensional evaluation of fatigue. Behavioral and cognitive dimensions of fatigue testing (e.g., open-field activity, forced-swim test) are also necessary to perform. The sample size (*n* = 6 per group) and exclusive use of male mice introduced statistical limitations, particularly regarding sex-specific responses. Based on previous studies, male mice are often selected as standard models in toxicological research due to their high metabolic stability and absence of reproductive cycle interference, which helps avoid hormonal fluctuations that could compromise data consistency. This is particularly important in long-term toxicity or pharmacodynamic evaluations. However, the lack of data from female mice inevitably hinders our comprehensive analysis of the effects of LB-Pro. Future experiments should evaluate dose-dependent effects in both male and female mice with larger cohorts to enhance statistical power.

### 3.3 Analysis of serum TNF-*α*, GSH-Px, NAD-MDH, and SOD concentrations

To explore metabolic differences among groups at the end of the experiment (after exhaustion test), orbital blood samples were collected from mice for measurement of serum TNF-*α* content, GSH-Px activity, NAD-MDH activity, and SOD activity.

In this experiment, CL group mice showed significantly lower serum TNF-α concentrations compared to CC group (6.36 ± 0.74 vs. 10.37 ± 1.84 pg./mL, *p* < 0.05), while CM and CH groups showed no significant differences compared to CC group (Figure 1C). This indicates that low-concentration LB-Pro reduces serum TNF-*α* levels in CRF mice. TNF-*α* is a cytokine/signal protein synthesized and released by immune cells, playing critical roles in immune responses, lipid metabolism regulation, inflammation, and oxidative stress (25). According to the findings of Yeung et al. (26), oral administration of probiotics significantly reduces serum TNF-*α* levels in 5-fluorouracil-treated mice. However, intriguingly, the CM and CH groups administered higher doses of LB-Pro did not exhibit a significant decrease in TNF-α levels. This discrepancy might be attributed to LB-Pro’s insensitivity to TNF-α regulation or the synergistic effects between *Lycium barbarum* components and probiotics. To better elucidate this phenomenon, future experiments could include an LB-Pro control group without chemotherapy treatment, test additional inflammatory cytokines (e.g., IL-6, IL-1β), or measure cytokine levels in the intestinal mucosa of mice. These approaches would provide a more comprehensive analysis of the mechanisms underlying LB-Pro’s anti-inflammatory effects on CRF in mice.

As shown in Figure 1C, CL group mice demonstrated significantly lower serum NAD-MDH activity compared to CC group (17.79 ± 2.32 vs. 22.53 ± 5.06 U/L, *p* < 0.05), while CM group showed significantly higher activity (27.95 ± 2.38 vs. 22.53 ± 5.06 U/L, *p* < 0.05). Serum NAD-MDH participates in metabolic pathways including the tricarboxylic acid cycle (TCA), amino acid metabolism, redox reactions, and gluconeogenesis (27, 28). Elevated NAD-MDH levels indicate increased energy demands (e.g., high-intensity metabolism or stress), while reduced levels suggest TCA cycle disruption or mitochondrial dysfunction which will potentially lead to lactate accumulation or redox imbalance.

Reduced NAD-MDH activity in CL group may relate to insufficient low-concentration LB-Pro intervention failing to effectively ameliorate chemotherapy-induced mitochondrial dysfunction. While the significant increase of NAD-MDH activity in CM group suggests medium-concentration LB-Pro might enhance mitochondrial respiratory chain complex activity (29) and TCA cycle flux (30) through gut microbiota and metabolite regulation (e.g., short-chain fatty acids), thereby improving energy supply efficiency. This aligns with CM group’s longest exhaustion time (4607.33 ± 1075.96 s), indicating mitochondrial function improvement as a potential anti-fatigue mechanism. No significant differences in CH group may reflect dose saturation effects or slight metabolic burdens.

CM and CH groups demonstrated significantly higher serum GSH-Px and SOD activities compared to CC group (*p* < 0.01 and *p* < 0.001) as shown in Figure 1C, consistent with exhaustion time results. GSH-Px is a selenium-dependent antioxidant enzyme critical for ROS scavenging, redox balance maintenance, and mitochondrial protection. Similarly, SOD can also alleviate oxidative damage. Under the influence of 5-fluorouracil, acute oxidative stimulation will occur in mice, and compensatory antioxidant reaction will increase GSH-Px and SOD activity. Bi et al. (12) demonstrated this, reporting that *Lycium barbarum* extract significantly elevated SOD activity in exercise-induced fatigue mouse models while reducing LDH levels and pro-inflammatory cytokine concentrations, thereby ameliorating oxidative stress-induced fatigue. Studies have also shown that the supplementation of exogenous antioxidants will also improve the vitality of both (31). The LB-Pros used in this study contains *Lycium barbarum* powder and fructooligosaccharides, etc. Polyphenols such as gallic acid, rutin and quercetin contained in *Lycium barbarum* can play an antioxidant role by scavenging free radicals and inhibiting lipid peroxidation. Moreover, LBP not only have prebiotic effect, but also promote the proliferation of *Bifidobacterium* and *Lactobacillus*, enhance the intestinal antioxidant barrier and indirectly enhance the host’s antioxidant capacity (32). It can also enhance the activity of intracellular antioxidant enzymes (such as CAT and SOD) by activating Nrf2/ARE signaling pathway (33). The results of this experiment are consistent with the aforementioned conclusions, indicating that the antioxidant properties of *Lycium barbarum* powder and fructooligosaccharides, as well as the probiotic components in LB-Pro, contribute to its efficacy in alleviating chemotherapy-induced CRF in mice.

Inevitably, we must acknowledge certain limitations of the current findings. First, the molecular mechanisms underlying these effects remain incompletely elucidated, as direct evidence for key regulatory pathways or mitochondrial functional analysis is lacking, which limits the mechanistic interpretation of LB-Pro’s anti-fatigue effects. For example, the inflammatory profile assessment was insufficient, as only TNF-*α* was measured, with no data on other critical cytokines or neuroinflammatory markers. Existing studies have shown that pro-inflammatory cytokines such as IL-6 and IL-1β may significantly influence the development of CRF through multiple pathways, including neuroimmune interactions, dysregulation of the HPA axis, central nervous system dysfunction, and mitochondrial impairment (1, 34, 35). Therefore, the absence of these data hinders a comprehensive understanding of the immune-metabolic interplay in CRF. Furthermore, their potential associations with the gut microbiota and metabolic axis remain undefined, which complicates the full explanation of LB-Pro’s synergistic mechanisms. To address these gaps, future research should prioritize multi-omics approaches to elucidate the molecular and metabolic underpinnings of LB-Pro’s effects. For instance, measuring mitochondrial function (e.g., ATP synthase activity, electron transport chain efficiency) and gene expression of key regulators (e.g., PGC-1α, Nrf2) would provide direct insights into energy metabolism and inflammation. Expanding cytokine profiling to include IL-6, IL-1β, and neuroimmune markers (e.g., serotonin, BDNF) would further clarify the mechanisms by which LB-Pro alleviates chemotherapy-induced CRF.

### 3.4 Effects of LB-Pros on gut microbiota

#### 3.4.1 Species composition of gut microbiota in mouse feces

**Figure 3:**
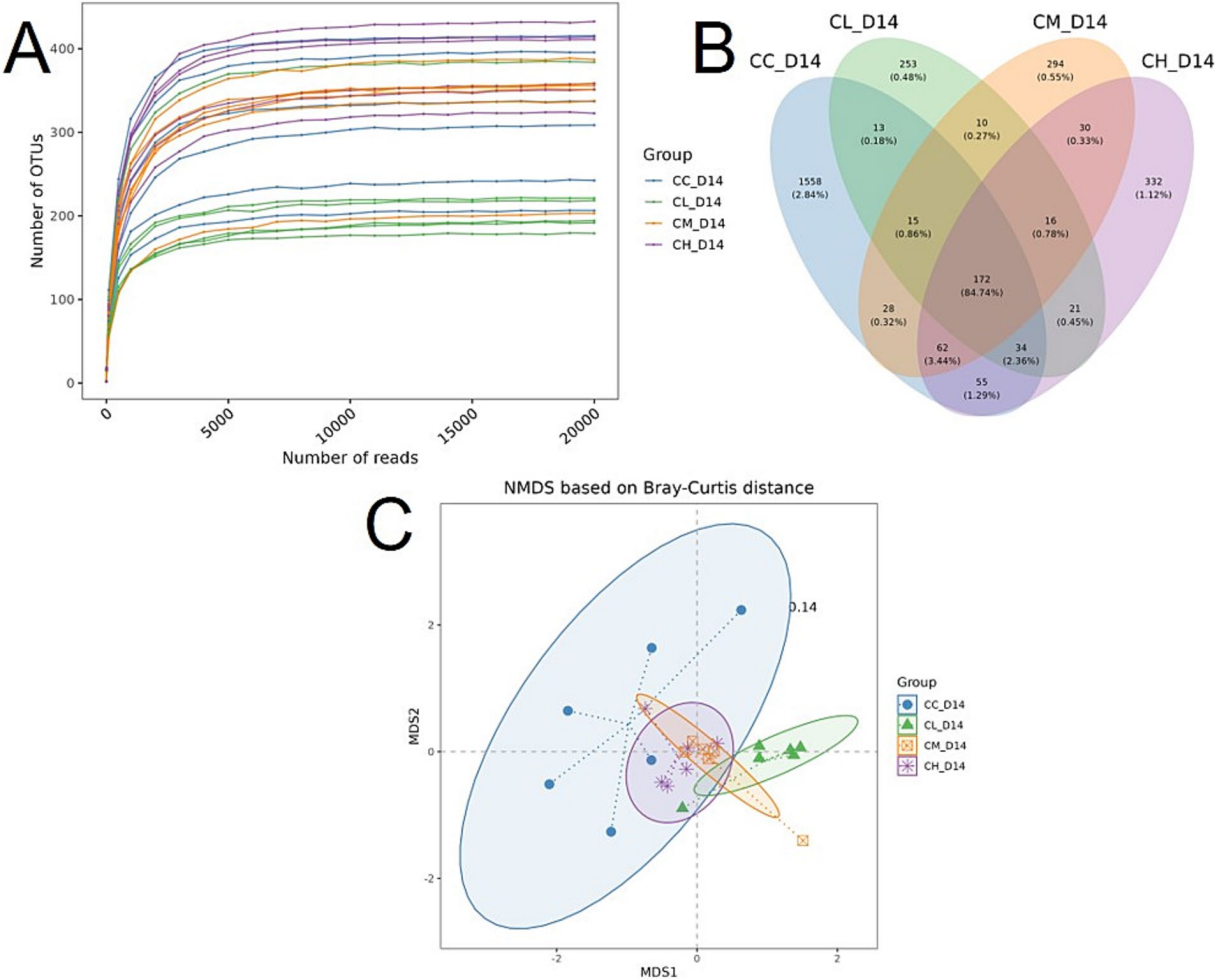
Microbial composition of fecal samples on Day 14. **(A)** Rarefaction curves showing species richness. **(B)** Venn plot of OTUs shared across groups. **(C)** NMDS plot based on Bray-Curtis distances with group ellipses. OTUs, operational taxonomic units; NMDS, Non-metric Multi-Dimensional Scaling; D14, fecal samples collected on Day 14; CC, Chemotherapy-Control group; CL, Chemotherapy-Low concentration LB-Pro group; CM, Chemotherapy-Medium concentration LB-Pro group; CH, Chemotherapy-High concentration LB-Pro group.

Microbial composition analysis of fecal samples from chemotherapy-induced CRF mice was performed via 16S rDNA sequencing. Prior to the intervention, we ensured that the baseline microbiota composition across groups was largely similar, including the relative abundance of high-abundance species, as well as alpha- and beta-diversity (see Supplementary Figures S1–S3). First, overall structural changes in gut microbiota were analyzed. As shown in Figure 3A, rarefaction curves were used to reflect species richness trends with sequencing depth in fecal samples. Each sample curve plateaued with increasing sequencing depth, indicating sufficient coverage to capture all species for subsequent bioinformatics analysis.

As shown in Figure 3B, the total number of shared operational taxonomic units (OTUs) across CC, CL, CM, and CH groups was 172, accounting for 84.74% of total OTU sequences. To evaluate compositional differences among groups, Non-metric Multi-Dimensional Scaling (NMDS) analysis was performed, projecting sample dissimilarities onto a 2D coordinate system. Figure 3C shows a stress value of 0.14. Compared to the control group (CC), mice treated with LB-Pros (CL, CM, and CH groups) exhibited distinct clustering patterns, indicating LB-Pros altered gut microbiota community structure in chemotherapy-induced CRF mice.

**Figure 4:**
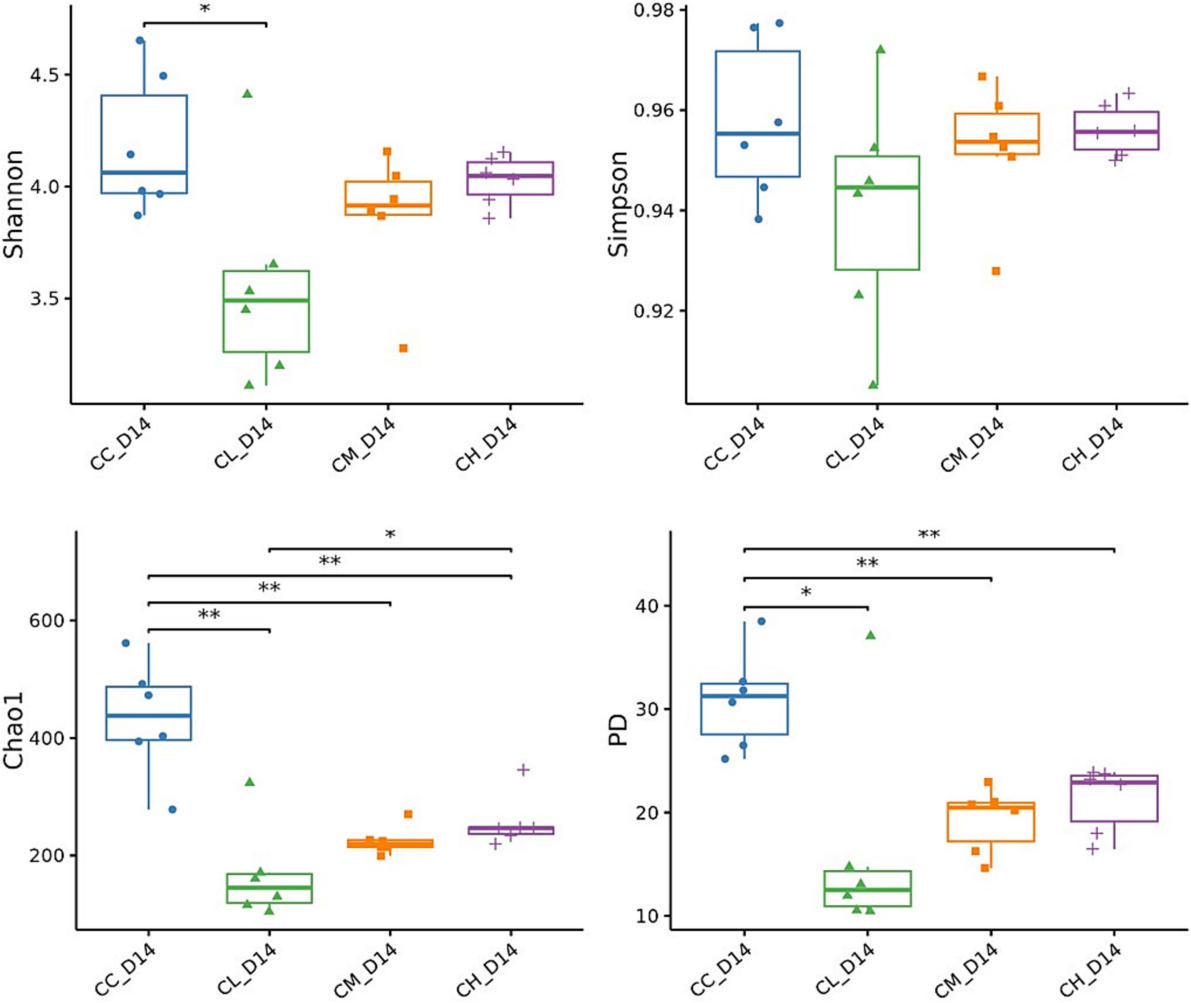
Alpha-diversity indices of gut microbiota on Day 14. Alpha-diversity indices (Shannon, Simpson, Chao 1, PD) for fecal samples. Shannon, quantifies species diversity and evenness; Simpson, reflects dominance, higher values mean lower diversity; Chao 1, estimates total species richness from rare species; PD, phylogenetic diversity, measures diversity based on evolutionary relationships; CC, Chemotherapy-Control group; CL, Chemotherapy-Low concentration LB-Pro group; CM, Chemotherapy-Medium concentration LB-Pro group; CH, Chemotherapy-High concentration LB-Pro group. *: *p* < 0.05; **: *p* < 0.01.

Then alpha-diversity indices (Shannon, Simpson, Chao 1, and PD index) were calculated to assess microbial richness, evenness, and diversity. As shown in Figure 4, CL group displayed significantly reduced Shannon index compared to CC group, while Chao 1 and PD indices were significantly decreased in CL, CM, and CH groups. These results demonstrate that LB-Pros altered gut microbiota structure in chemotherapy-induced CRF mice.

Next, to visualize high-abundance species, stacked bar charts were generated at phylum and genus levels. As shown in Figure 2, dominant phyla across all groups included *Bacteroidetes*, *Firmicutes*, *Proteobacteria*, and *Actinobacteria* and so on. Dominant genera included *Bacteroides*, *Lachnospiraceae NK4A136 group*, *Alloprevotella*, *Lactobacillus* and so on.

**Figure 5:**
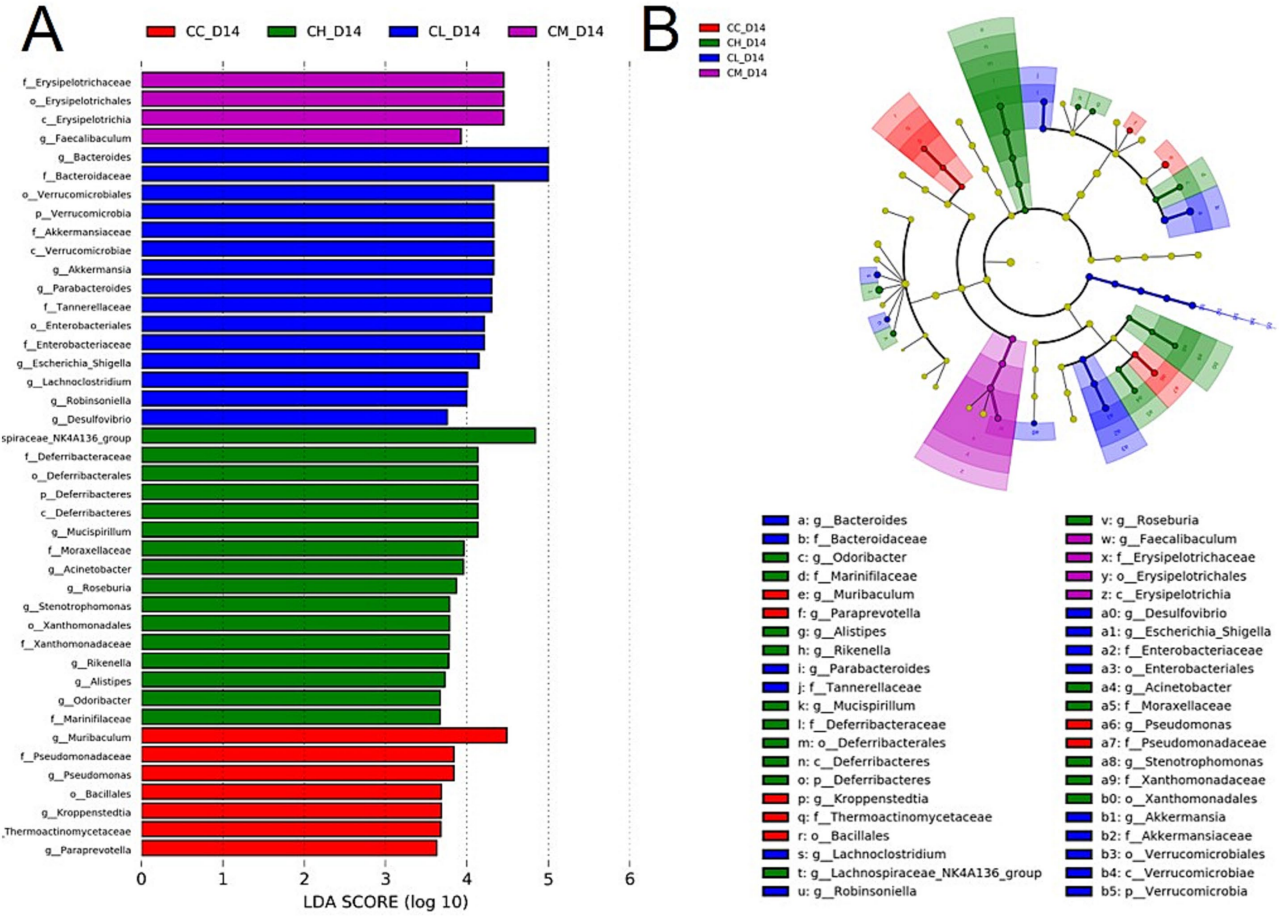
LEfSe analysis of gut microbiota on Day 14. **(A)** LDA scores of discriminative taxa. Show the species significantly enriched in each group and their importance. **(B)** Cladogram showing taxonomic relationships. It is used to show the distribution law of phylogenetic relationships of species that play an important role in each group of samples. LDA, linear discriminant analysis. CC, Chemotherapy-Control group; CL, Chemotherapy-Low concentration LB-Pro group; CM, Chemotherapy-Medium concentration LB-Pro group; CH, Chemotherapy-High concentration LB-Pro group.

Given the significant fatigue alleviation effects in CM and CH groups, LEfSe analysis was conducted to further determine the characteristic microorganisms in CM group and CH group. Figure 5 shows CM group contained 4 discriminative taxa: *Faecalibaculum* (genus level) and *Erysipelotrichia-Erysipelotrichales-Erysipelotrichaceae* (class-order-family level). CH group contained 16 discriminative taxa, primarily *Deferribacteres-Deferribacteres-Deferribacterales-Deferribacteraceae* (phylum-class-order-family level), *Roseburia* (genus level), *Rikenella* (genus level), and *Alistipes* (genus level). These bacteria primarily participate in metabolic processes such as short-chain fatty acid (SCFA) metabolism (36, 37), bile acid metabolism (38), and metal redox reactions (39) in mice.

Combined analysis suggests appropriate probiotic concentrations alleviate chemotherapy-induced fatigue through gut microbiota-host metabolic axis regulation. One of the potential mechanisms involves enhanced SCFA metabolism, as CM group-enriched *Erysipelotrichaceae* and CH group-enriched *Roseburia* are known SCFA (such as butyrate, propionate) producers. SCFAs may improve gut barrier function, reduce inflammatory cytokine release, and modulate central nervous system energy metabolism/fatigue perception via G protein-coupled receptors (GPCRs) activation (40) or histone deacetylase (HDAC) inhibition (41).

However, the proposed stimulatory effect of LB-Pro on SCFAs remains a hypothesis derived from the observed shifts in gut microbiota composition, this association lacks direct experimental validation through quantification of SCFAs (e.g., acetic acid, propionic acid, butyric acid) or targeted metabolomic profiling. Their metabolic contributions to SCFA synthesis remain speculative without biochemical measurements. This limitation underscores the need for future studies to integrate SCFA quantification using advanced techniques such as gas chromatography–mass spectrometry (GC–MS) or liquid chromatography-mass spectrometry (LC–MS), alongside functional metabolomic analyses, to establish a mechanistic connection between microbial community dynamics and host metabolic outcomes. Such investigations would strengthen the evidence base for LB-Pro’s role in modulating the gut microbiota-metabolic axis and its anti-fatigue effects.

#### 3.4.2 Functional prediction of gut microbiota communities in mouse feces

**Figure 6:**
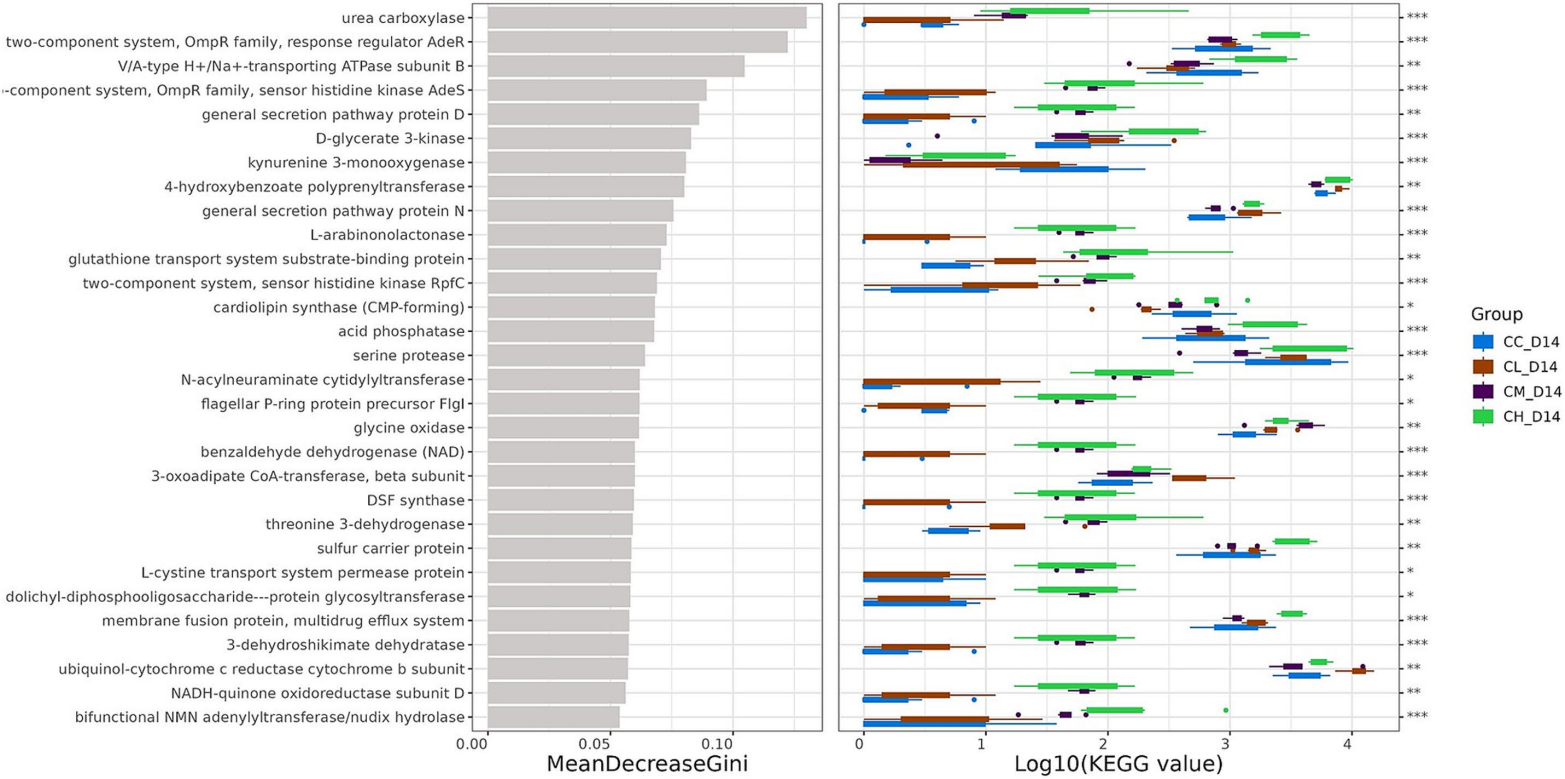
Functional prediction of gut microbiota on Day 14. Random forest analysis of microbial functional categories. The abscissa of the left graph is the average decreasing value of Gini index, and the ordinate is the classification information of groups, while the right graph is the box graph of group abundance of different groups. Categories without accurate classification information were not analyzed. From top to bottom, the importance of affecting grouping decreases in turn. Gini index, used to evaluate the importance of each Feature (species here), and the faster the Gini index drops, the greater the importance of species in inter-group classification; KEGG, Kyoto Encyclopedia of Genes and Genomes; CC, Chemotherapy-Control group; CL, Chemotherapy-Low concentration LB-Pro group; CM, Chemotherapy-Medium concentration LB-Pro group; CH, Chemotherapy-High concentration LB-Pro group. ^*^p < 0.05; ^**^p < 0.01; ^***^p < 0.001.

This study utilized PICRUSt2 functional prediction tool combined with Random Forest machine learning analysis to predict fecal microbial functions. As shown in Figure 6, considering the significant fatigue alleviation effects in CM and CH groups compared to CC and CL groups, functional categories with notably higher abundances in CM and CH groups were selected. These primarily involved metabolic processes including:

1. Enhanced energy metabolism and mitochondrial function: NADH-quinone oxidoreductase subunit D directly participates in mitochondrial electron transport chain to promote ATP synthesis and energy supply efficiency (42). And threonine 3-dehydrogenase catalyzes threonine metabolism to generate pyruvate and acetyl-CoA, providing substrates for TCA cycle (43). Benzaldehyde dehydrogenase (NAD) participates in aldehyde oxidation to maintain NAD+/NADH balance and optimize mitochondrial oxidative phosphorylation efficiency.
2. Activated antioxidant defense system: L-cystine transport system permease protein facilitates cysteine uptake to support glutathione (GSH) biosynthesis (44). And benzaldehyde dehydrogenase (NAD) scavenges aldehyde oxidation products (e.g., lipid peroxidation end products) to reduce ROS accumulation.
3. Gut barrier and immune regulation: General secretion pathway protein D participates in bacterial outer membrane protein secretion, potentially modulating probiotic colonization or competitive inhibition of pathogens to maintain gut barrier integrity (45). DSF synthase regulates bacterial quorum sensing, influencing microbial symbiosis and host immune responses (46). N-acetylneuraminic cytidylyltransferase participates in sialic acid metabolism, possibly affecting intestinal immune tolerance by modifying mucus layer components.
4. Regulation of signaling pathway: 3-dehydroshikimate dehydratase participates in aromatic amino acid metabolism, potentially regulating tryptophan-kynurenine pathway to influence neurotransmitter synthesis or inflammation (47). Dolichyl-diphosphooligosaccharide--protein glycosyltransferase mediates protein glycosylation (48), affecting host cell signaling or immunoglobulin function.

These functional predictions suggest that medium- and high-concentration LB-Pros may alleviate CRF through multi-level metabolic regulation, providing new insights into gut microbiota-host interactions in chemotherapy-induced CRF in C57BL/6NCr mice. However, these functional predictions are based solely on microbial composition data and computational modeling, without direct experimental validation of key metabolic pathways or enzymatic activities. The proposed mechanisms remain hypothetical and require further investigation using targeted molecular and biochemical approaches. For instance, future studies should employ qPCR to quantify the expression of genes associated with energy metabolism and antioxidant defense (e.g., *nad1, ldhA, gsh1*), providing molecular evidence for the observed functional shifts. At the tissue level, future investigations should integrate histological analyses of gut barrier integrity (e.g., tight junction proteins, epithelial damage) and tissue pathology of muscles/livers (e.g., mitochondrial morphology via electron microscopy) to connect microbial and metabolic alterations to tissue-level outcomes. Additionally, integrating multi-omics approaches—combining metagenomic, transcriptomic, and proteomic data—will help unravel causal relationships between microbial functions and the alleviation of chemotherapy-induced CRF. These steps are essential to validate the proposed mechanisms, refine the therapeutic framework of LB-Pro, and advance its clinical translation for managing CRF through microbiota-targeted strategies.

On the other hand, the LB-Pro used in this study combines prebiotics and probiotics, and although their combined effects are promising, analyzing the contribution of each component can help us better understand the underlying mechanisms and optimize therapeutic efficacy. To clarify the individual roles, future studies should adopt single-component or factorial designs to isolate the effects of each ingredient. Metabolomic and transcriptomic analyses could further elucidate strain-specific. Such investigations will refine the therapeutic potential of this formulation and guide its application in personalized interventions for diseases like CRF.

## 4 Conclusion

This study focused on CRF, systematically investigating the mechanisms of different concentrations of *Lycium barbarum* and LB-Pros in alleviating fatigue symptoms and their association with the gut microbiota-metabolic axis using a chemotherapy-induced CRF C57BL/6NCr mouse model. Results showed that medium-concentration (100 mg/kg) and high-concentration (500 mg/kg) LB-Pros significantly enhanced serum GSH-Px and SOD activities in mice, effectively alleviating fatigue symptoms. This likely occurred through mechanisms including enhanced mitochondrial energy metabolism and antioxidant defense systems to mitigate chemotherapy-induced oxidative stress damage. Gut microbiota analysis indicated that medium- and high-concentration LB-Pros may regulate the gut microbiota-metabolic axis by regulating SCFAs metabolism, enhancing mitochondrial energy metabolism, activating antioxidant defense systems, and modulating gut barrier function, thereby effectively alleviating CRF. And the medium-concentration intervention demonstrated the most significant effects, while the high-concentration group showed slightly reduced efficacy, suggesting potential dose saturation effects or metabolic burdens at excessive concentrations. However, this study has several limitations, including incomplete elucidation of molecular mechanisms, lack of histological validation, and a small sample size. Future research should expand the scope of investigations by integrating metabolomics and histological analyses, increasing sample size, and conducting more in-depth studies to experimentally validate the proposed mechanisms and refine the therapeutic framework of LB-Pro.

This study offers novel insights into microecological and traditional Chinese medicine interventions for CRF. However, the complexity of underlying mechanisms and inter-individual variability necessitate further in-depth research and interdisciplinary collaboration to facilitate the translation of basic findings into clinical applications.

## Data Availability

The original contributions presented in the study are included in the article/Supplementary material, further inquiries can be directed to the corresponding authors.
