# Supplementary material for: Lycium barbarum-probiotic synergy alleviates chemotherapy-induced cancer-related fatigue via gut microbiota-metabolic axis regulation in mice
Source: Front Nutr. 2025 Jul 2;12:1613132. doi: 10.3389/fnut.2025.1613132 (PMC12263555; doi:10.3389/fnut.2025.1613132)
Supplement: Supplementary file 1 [file Supplementary_file_1.zip › Supplementary Figures.DOCX]

Supplementary Material

**
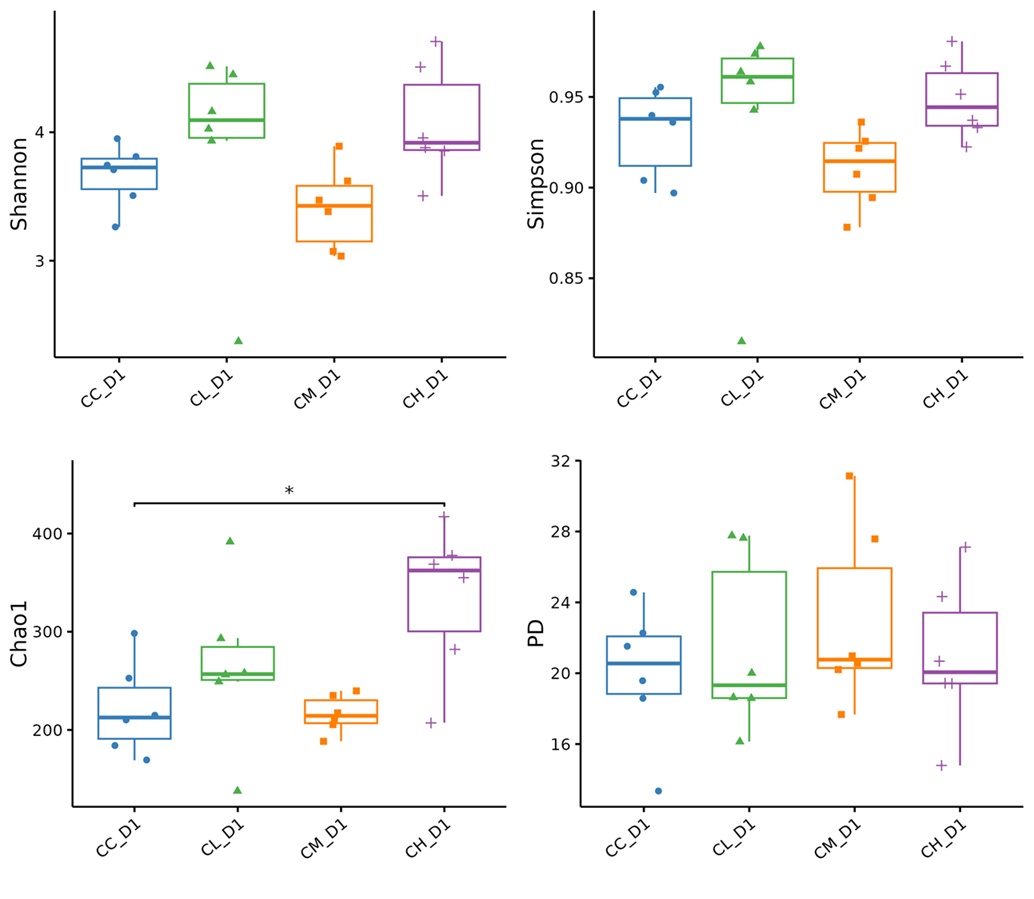
**1.

**Figure 1S. Alpha-Diversity Indices of Gut Microbiota on Day 1.** Alpha-diversity indices (Shannon, Simpson, Chao 1, PD) for fecal samples. Note: Shannon, quantifies species diversity and evenness; Simpson, reflects dominance, higher values mean lower diversity; Chao 1, estimates total species richness from rare species; PD, phylogenetic diversity, measures diversity based on evolutionary relationships; CC, Chemotherapy-Control group; CL, Chemotherapy-Low concentration LB-Pro group; CM, Chemotherapy-Medium concentration LB-Pro group; CH, Chemotherapy-High concentration LB-Pro group.


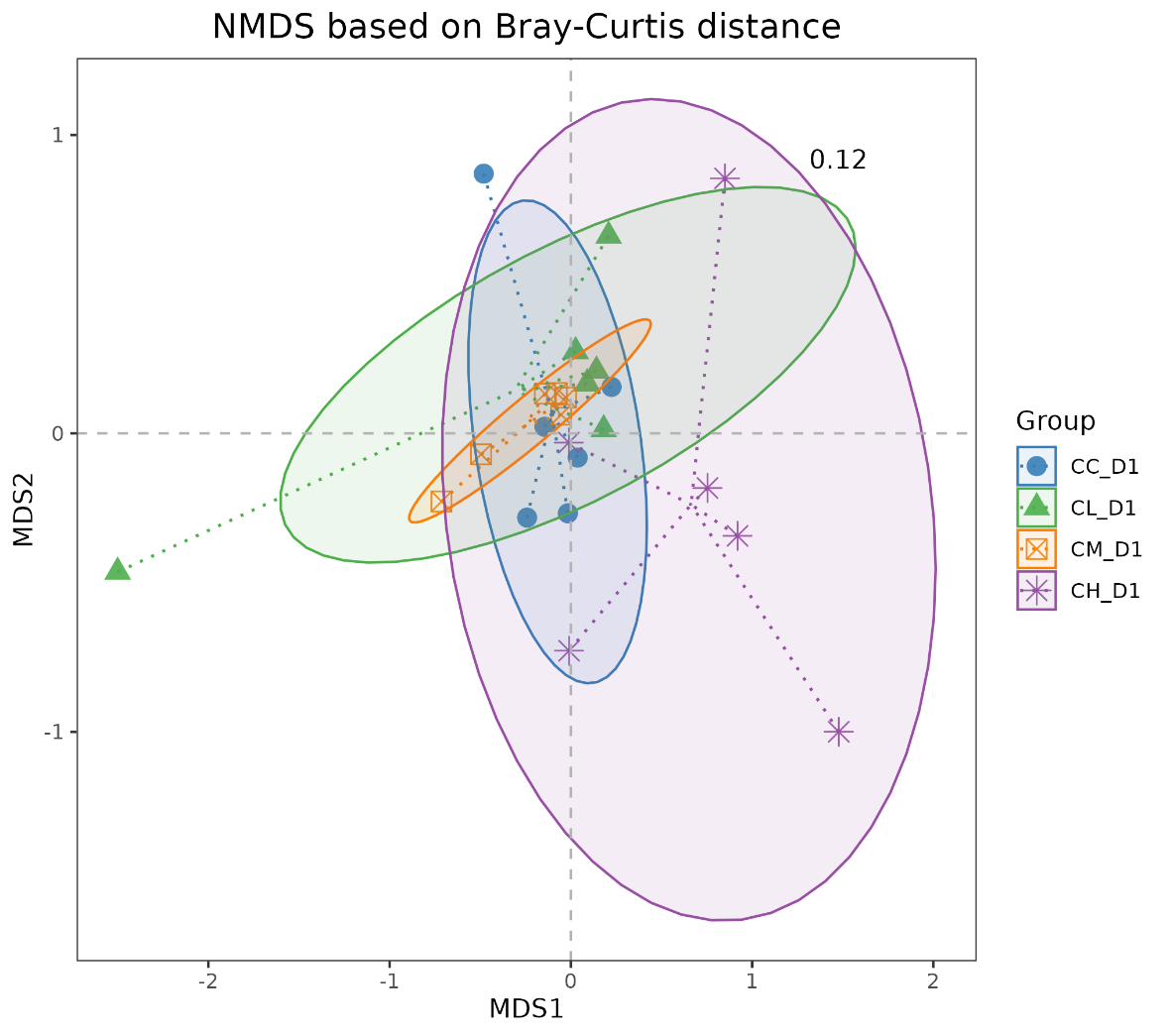
2.

**Figure 2S. NMDS plot based on Bray-Curtis distances with group ellipses of Fecal Samples on Day 1.** Note: NMDS, Non-metric Multi-Dimensional Scaling; D1, fecal samples collected on Day 1; CC, Chemotherapy-Control group; CL, Chemotherapy-Low concentration LB-Pro group; CM, Chemotherapy-Medium concentration LB-Pro group; CH, Chemotherapy-High concentration LB-Pro group.


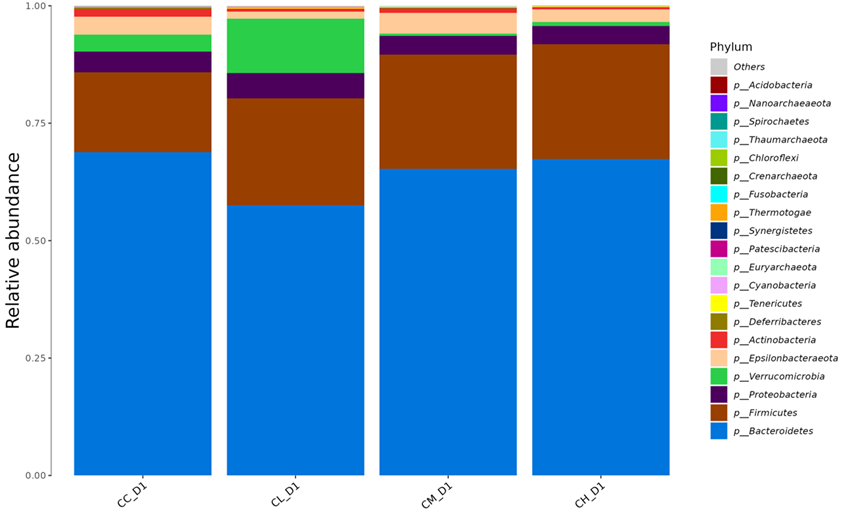
3.

**Figure 3S. Phylum-level relative abundance of Gut Microbiota on Day 1.** Note: *p_*, phylum level; CC, Chemotherapy-Control group; CL, Chemotherapy-Low concentration LB-Pro group; CM, Chemotherapy-Medium concentration LB-Pro group; CH, Chemotherapy-High concentration LB-Pro group.
