# Supplementary material for: Lycium barbarum-probiotic synergy alleviates chemotherapy-induced cancer-related fatigue via gut microbiota-metabolic axis regulation in mice
Source: Front Nutr. 2025 Jul 2;12:1613132. doi: 10.3389/fnut.2025.1613132 (PMC12263555; doi:10.3389/fnut.2025.1613132)
Supplement: Supplementary file 1 [file Supplementary_file_1.zip › Raw Data 2.ZIP!/Gut microbiota analysis data-2/罗宁生物扩增子测序报告/06.TaxaVariation/Diff_taxa_kw/Family/Diff_taxa_kw_Family_8_boxplot.pdf]

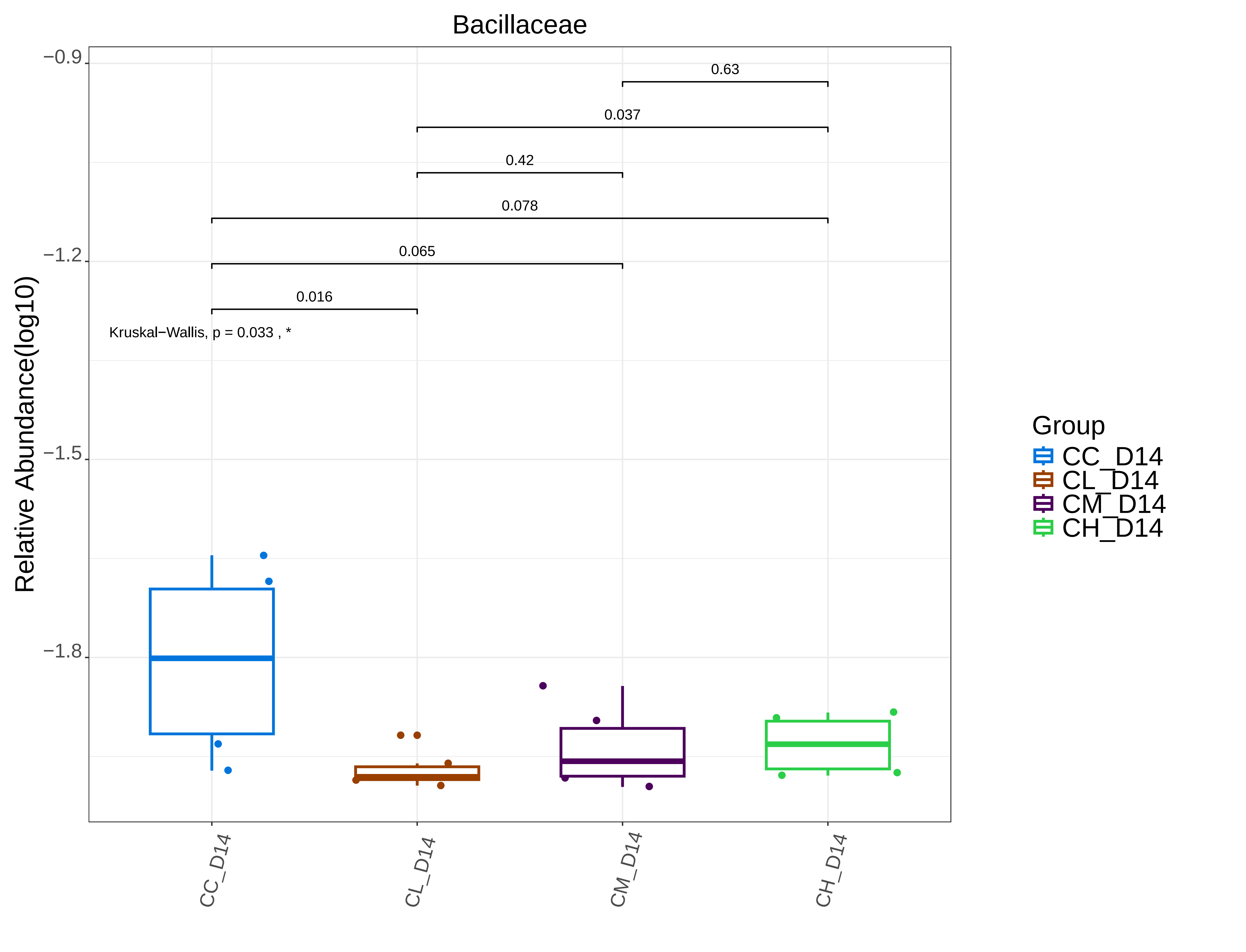

Bacillaceae
0.63
0.037
0.42
0.078
0.065
0.016
Kruskal−Wallis, p = 0.033 , *
−0.9
−1.2
−1.5
−1.8
Relative Abundance(log10)
CC_D14
CL_D14
CM_D14
CH_D14
Group
CC_D14
CL_D14
CM_D14
CH_D14
