# Supplementary material for: Lycium barbarum-probiotic synergy alleviates chemotherapy-induced cancer-related fatigue via gut microbiota-metabolic axis regulation in mice
Source: Front Nutr. 2025 Jul 2;12:1613132. doi: 10.3389/fnut.2025.1613132 (PMC12263555; doi:10.3389/fnut.2025.1613132)
Supplement: Supplementary file 1 [file Supplementary_file_1.zip › Raw Data 2.ZIP!/Gut microbiota analysis data-2/罗宁生物扩增子测序报告/06.TaxaVariation/Diff_taxa_kw/Genus/Diff_taxa_kw_Genus_heatmap_Group.pdf]

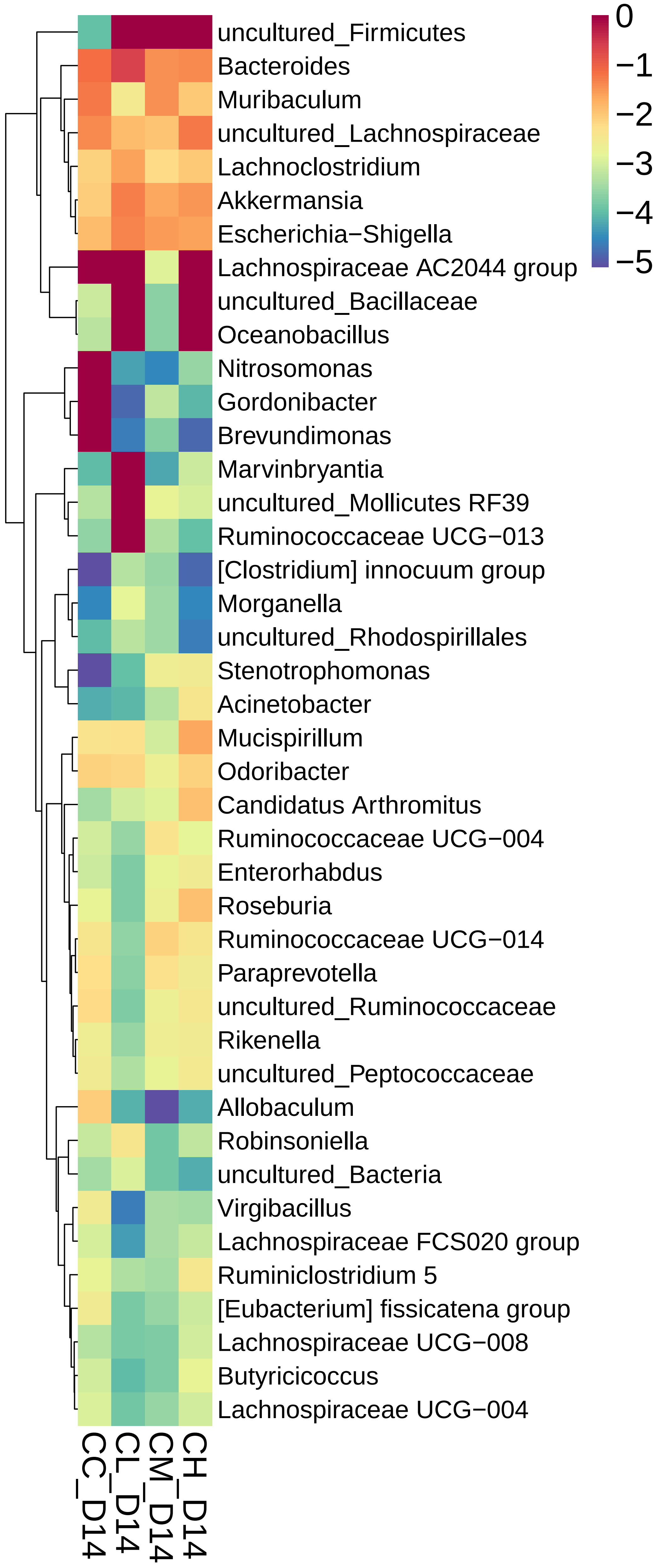
0
−1
−2
−3
−4
−5
uncultured_Firmicutes
Bacteroides
Muribaculum
uncultured_Lachnospiraceae
Lachnoclostridium
Akkermansia
Escherichia−Shigella
Lachnospiraceae AC2044 group
uncultured_Bacillaceae
Oceanobacillus
Nitrosomonas
Gordonibacter
Brevundimonas
Marvinbryantia
uncultured_Mollicutes RF39
Ruminococcaceae UCG−013
[Clostridium] innocuum group
Morganella
uncultured_Rhodospirillales
Stenotrophomonas
Acinetobacter
Mucispirillum
Odoribacter
Candidatus Arthromitus
Ruminococcaceae UCG−004
Enterorhabdus
Roseburia
Ruminococcaceae UCG−014
Paraprevotella
uncultured_Ruminococcaceae
Rikenella
uncultured_Peptococcaceae
Allobaculum
Robinsoniella
uncultured_Bacteria
Virgibacillus
Lachnospiraceae FCS020 group
Ruminiclostridium 5
[Eubacterium] fissicatena group
Lachnospiraceae UCG−008
Butyricicoccus
Lachnospiraceae UCG−004
CC_D14
CL_D14
CM_D14
CH_D14
