# Supplementary material for: Lycium barbarum-probiotic synergy alleviates chemotherapy-induced cancer-related fatigue via gut microbiota-metabolic axis regulation in mice
Source: Front Nutr. 2025 Jul 2;12:1613132. doi: 10.3389/fnut.2025.1613132 (PMC12263555; doi:10.3389/fnut.2025.1613132)
Supplement: Supplementary file 1 [file Supplementary_file_1.zip › Raw Data 2.ZIP!/Gut microbiota analysis data-2/罗宁生物扩增子测序报告/06.TaxaVariation/Diff_taxa_kw/Order/Diff_taxa_kw_Order_heatmap_Group.pdf]

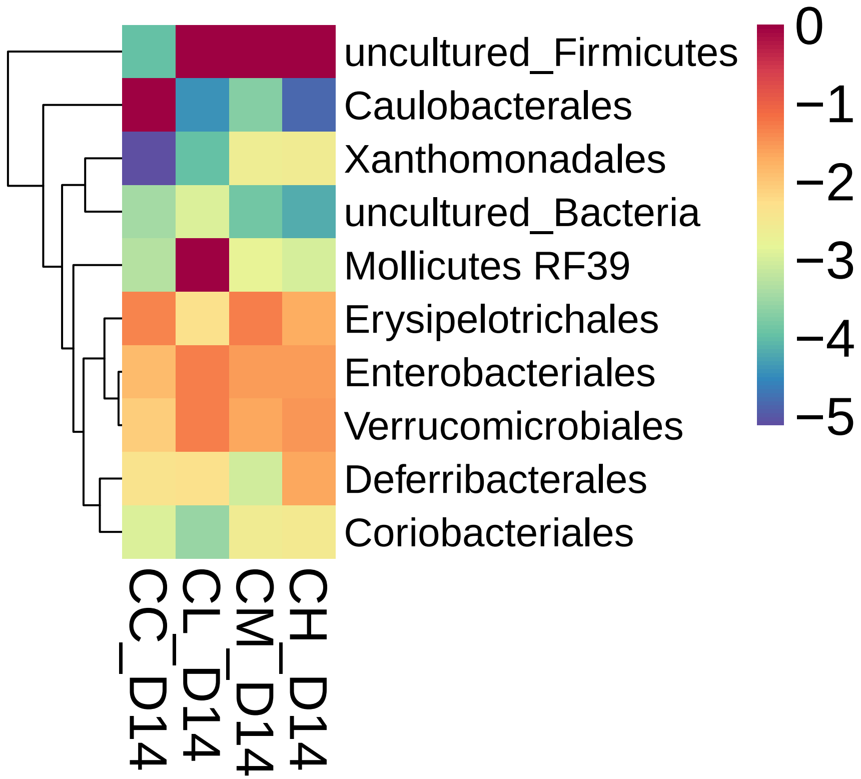
uncultured_Firmicutes
Caulobacterales
Xanthomonadales
uncultured_Bacteria
Mollicutes RF39
Erysipelotrichales
Enterobacteriales
Verrucomicrobiales
Deferribacterales
Coriobacteriales
CC_D14
CL_D14
CM_D14
CH_D14
0
−1
−2
−3
−4
−5
