# Supplementary material for: Lycium barbarum-probiotic synergy alleviates chemotherapy-induced cancer-related fatigue via gut microbiota-metabolic axis regulation in mice
Source: Front Nutr. 2025 Jul 2;12:1613132. doi: 10.3389/fnut.2025.1613132 (PMC12263555; doi:10.3389/fnut.2025.1613132)
Supplement: Supplementary file 1 [file Supplementary_file_1.zip › Raw Data 2.ZIP!/Gut microbiota analysis data-2/罗宁生物扩增子测序报告/06.TaxaVariation/Diff_taxa_kw/Phylum/Diff_taxa_kw_Phylum_heatmap.pdf]

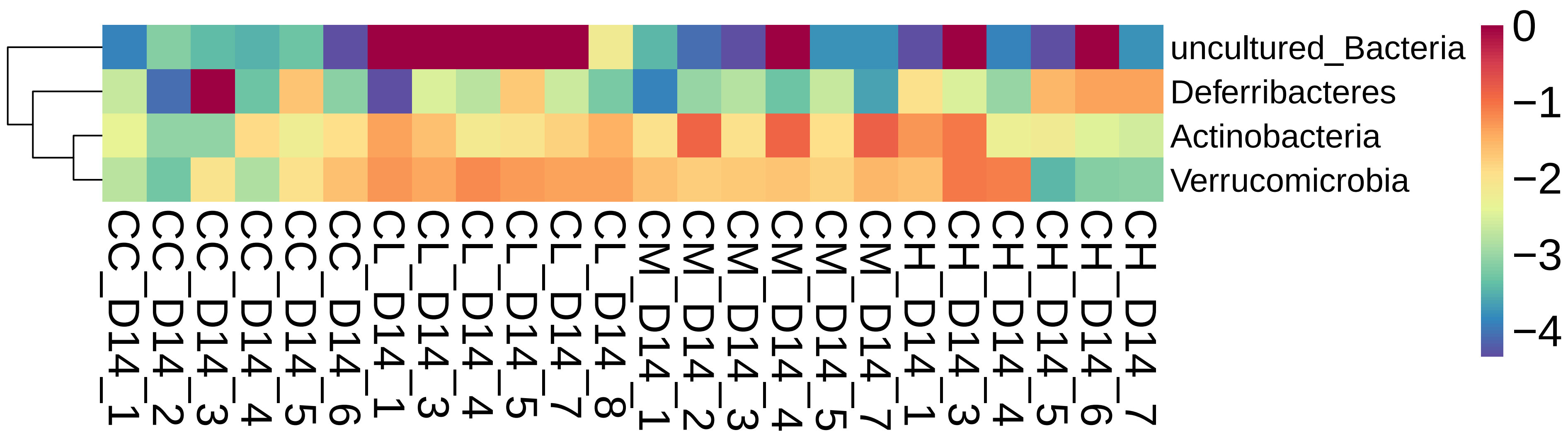
uncultured_Bacteria
Deferribacteres
Actinobacteria
Verrucomicrobia
CC_D14_1
CC_D14_2
CC_D14_3
CC_D14_4
CC_D14_5
CC_D14_6
CL_D14_1
CL_D14_3
CL_D14_4
CL_D14_5
CL_D14_7
CL_D14_8
CM_D14_1
CM_D14_2
CM_D14_3
CM_D14_4
CM_D14_5
CM_D14_7
CH_D14_1
CH_D14_3
CH_D14_4
CH_D14_5
CH_D14_6
CH_D14_7
0
−1
−2
−3
−4
