# Supplementary material for: Lycium barbarum-probiotic synergy alleviates chemotherapy-induced cancer-related fatigue via gut microbiota-metabolic axis regulation in mice
Source: Front Nutr. 2025 Jul 2;12:1613132. doi: 10.3389/fnut.2025.1613132 (PMC12263555; doi:10.3389/fnut.2025.1613132)
Supplement: Supplementary file 1 [file Supplementary_file_1.zip › Raw Data 2.ZIP!/Gut microbiota analysis data-2/罗宁生物扩增子测序报告/06.TaxaVariation/Diff_taxa_kw/Species/Diff_taxa_kw_Species_heatmap.pdf]

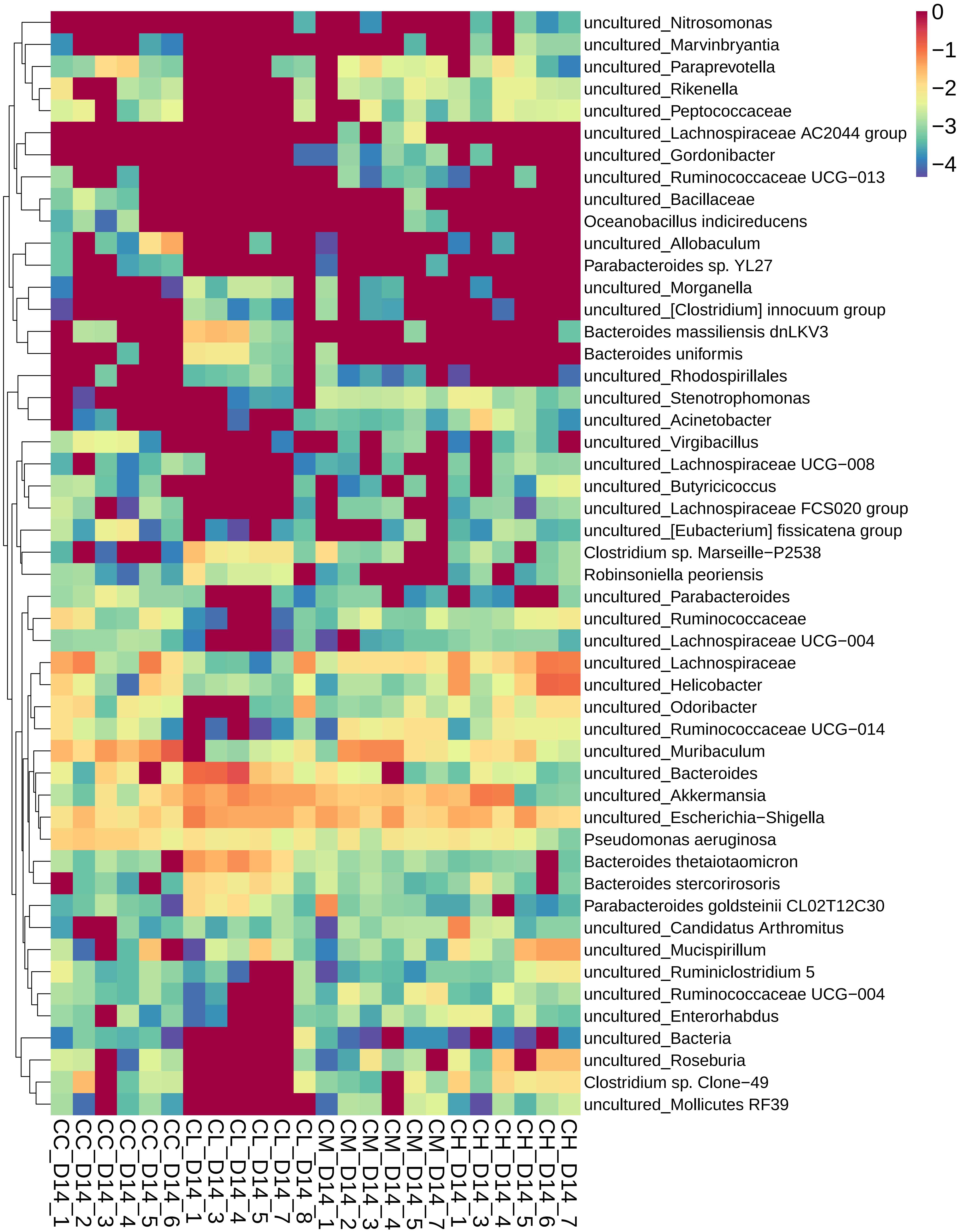
0
−1
−2
−3
−4
uncultured_Nitrosomonas
uncultured_Marvinbryantia
uncultured_Paraprevotella
uncultured_Rikenella
uncultured_Peptococcaceae
uncultured_Lachnospiraceae AC2044 group
uncultured_Gordonibacter
uncultured_Ruminococcaceae UCG−013
uncultured_Bacillaceae
Oceanobacillus indicireducens
uncultured_Allobaculum
Parabacteroides sp. YL27
uncultured_Morganella
uncultured_[Clostridium] innocuum group
Bacteroides massiliensis dnLKV3
Bacteroides uniformis
uncultured_Rhodospirillales
uncultured_Stenotrophomonas
uncultured_Acinetobacter
uncultured_Virgibacillus
uncultured_Lachnospiraceae UCG−008
uncultured_Butyricicoccus
uncultured_Lachnospiraceae FCS020 group
uncultured_[Eubacterium] fissicatena group
Clostridium sp. Marseille−P2538
Robinsoniella peoriensis
uncultured_Parabacteroides
uncultured_Ruminococcaceae
uncultured_Lachnospiraceae UCG−004
uncultured_Lachnospiraceae
uncultured_Helicobacter
uncultured_Odoribacter
uncultured_Ruminococcaceae UCG−014
uncultured_Muribaculum
uncultured_Bacteroides
uncultured_Akkermansia
uncultured_Escherichia−Shigella
Pseudomonas aeruginosa
Bacteroides thetaiotaomicron
Bacteroides stercorirosoris
Parabacteroides goldsteinii CL02T12C30
uncultured_Candidatus Arthromitus
uncultured_Mucispirillum
uncultured_Ruminiclostridium 5
uncultured_Ruminococcaceae UCG−004
uncultured_Enterorhabdus
uncultured_Bacteria
uncultured_Roseburia
Clostridium sp. Clone−49
uncultured_Mollicutes RF39
CC_D14_1
CC_D14_2
CC_D14_3
CC_D14_4
CC_D14_5
CC_D14_6
CL_D14_1
CL_D14_3
CL_D14_4
CL_D14_5
CL_D14_7
CL_D14_8
CM_D14_1
CM_D14_2
CM_D14_3
CM_D14_4
CM_D14_5
CM_D14_7
CH_D14_1
CH_D14_3
CH_D14_4
CH_D14_5
CH_D14_6
CH_D14_7
