# Supplementary material for: Lycium barbarum-probiotic synergy alleviates chemotherapy-induced cancer-related fatigue via gut microbiota-metabolic axis regulation in mice
Source: Front Nutr. 2025 Jul 2;12:1613132. doi: 10.3389/fnut.2025.1613132 (PMC12263555; doi:10.3389/fnut.2025.1613132)
Supplement: Supplementary file 1 [file Supplementary_file_1.zip › Raw Data 2.ZIP!/Gut microbiota analysis data-2/罗宁生物扩增子测序报告/06.TaxaVariation/Diff_taxa_kw/Species/Diff_taxa_kw_Species_heatmap_Group.pdf]

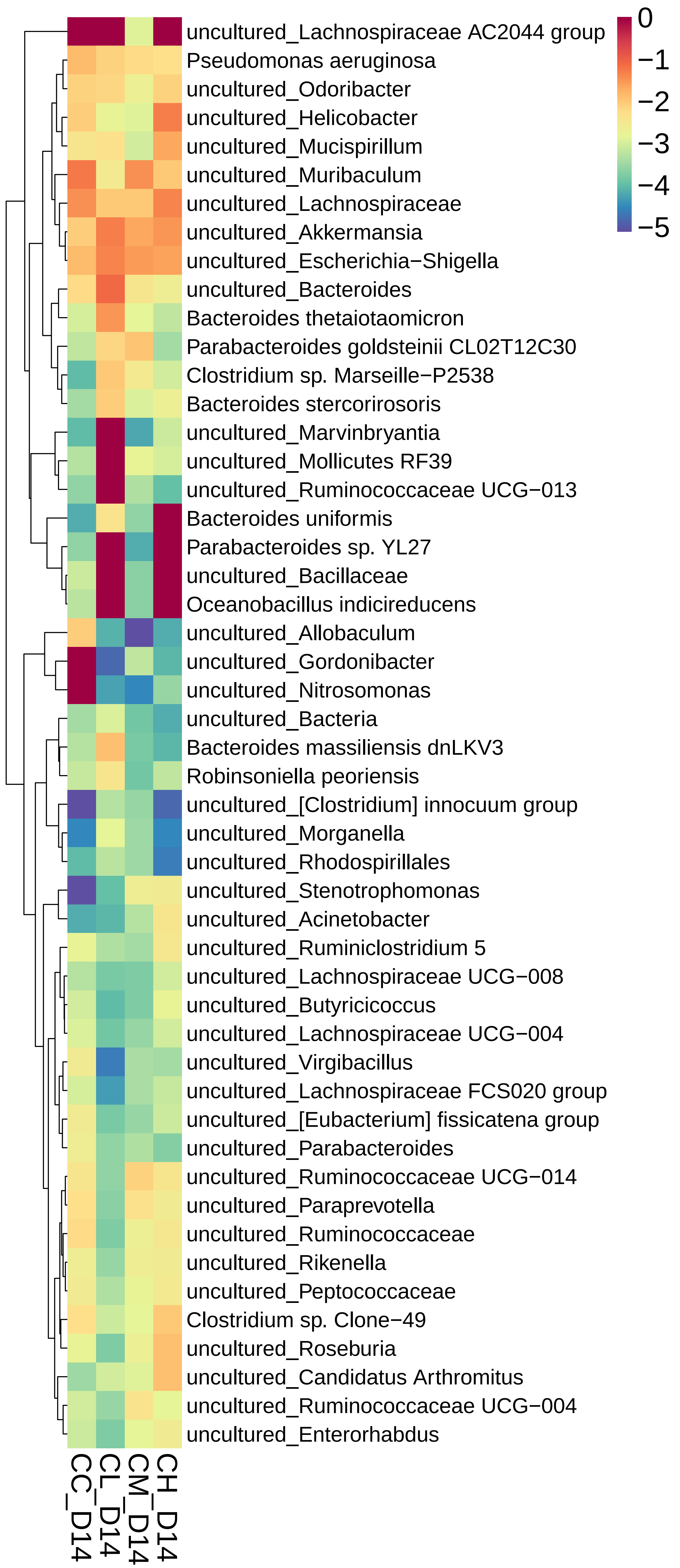

0
−1
−2
−3
−4
−5
uncultured_Lachnospiraceae AC2044 group
Pseudomonas aeruginosa
uncultured_Odoribacter
uncultured_Helicobacter
uncultured_Mucispirillum
uncultured_Muribaculum
uncultured_Lachnospiraceae
uncultured_Akkermansia
uncultured_Escherichia−Shigella
uncultured_Bacteroides
Bacteroides thetaiotaomicron
Parabacteroides goldsteinii CL02T12C30
Clostridium sp. Marseille−P2538
Bacteroides stercorirosoris
uncultured_Marvinbryantia
uncultured_Mollicutes RF39
uncultured_Ruminococcaceae UCG−013
Bacteroides uniformis
Parabacteroides sp. YL27
uncultured_Bacillaceae
Oceanobacillus indicireducens
uncultured_Allobaculum
uncultured_Gordonibacter
uncultured_Nitrosomonas
uncultured_Bacteria
Bacteroides massiliensis dnLKV3
Robinsoniella peoriensis
uncultured_[Clostridium] innocuum group
uncultured_Morganella
uncultured_Rhodospirillales
uncultured_Stenotrophomonas
uncultured_Acinetobacter
uncultured_Ruminiclostridium 5
uncultured_Lachnospiraceae UCG−008
uncultured_Butyricicoccus
uncultured_Lachnospiraceae UCG−004
uncultured_Virgibacillus
uncultured_Lachnospiraceae FCS020 group
uncultured_[Eubacterium] fissicatena group
uncultured_Parabacteroides
uncultured_Ruminococcaceae UCG−014
uncultured_Paraprevotella
uncultured_Ruminococcaceae
uncultured_Rikenella
uncultured_Peptococcaceae
Clostridium sp. Clone−49
uncultured_Roseburia
uncultured_Candidatus Arthromitus
uncultured_Ruminococcaceae UCG−004
uncultured_Enterorhabdus
CC_D14
CL_D14
CM_D14
CH_D14
