# Supplementary material for: Lycium barbarum-probiotic synergy alleviates chemotherapy-induced cancer-related fatigue via gut microbiota-metabolic axis regulation in mice
Source: Front Nutr. 2025 Jul 2;12:1613132. doi: 10.3389/fnut.2025.1613132 (PMC12263555; doi:10.3389/fnut.2025.1613132)
Supplement: Supplementary file 1 [file Supplementary_file_1.zip › Raw Data 2.ZIP!/Original data description.docx]

1. “Measurement datas .xlsx” contains the original data of mouse weight, running time, serum TNF-α content, GSH-Px activity, NAD-MDH activity and SOD activity.
2. The folder “Gut microbiota analysis data” contains the original data of gut microbiota in the feces of mice on the 14th day. And the gut microbiota analysis was performed by Rhonin Biosciences Co., Ltd. (Chengdu, China), which generated raw sequencing data and analysis results in Chinese.
3. Because the original data “Gut microbiota analysis data” has exceeded the limit of 30M, we must divide it into two parts. “Gut microbiota analysis data-1” and “Gut microbiota analysis data-2” are placed in two compressed packages respectively.
