# Supplementary material for: Lycium barbarum-probiotic synergy alleviates chemotherapy-induced cancer-related fatigue via gut microbiota-metabolic axis regulation in mice
Source: Front Nutr. 2025 Jul 2;12:1613132. doi: 10.3389/fnut.2025.1613132 (PMC12263555; doi:10.3389/fnut.2025.1613132)
Supplement: Supplementary file 1 [file Supplementary_file_1.zip › Raw Data 1.ZIP!/Gut microbiota analysis data-1/罗宁生物扩增子测序报告/05.FunctionPrediction/FAPROTAX/Statistic/PCA/FAPROTAX_PCA_Group.pdf]

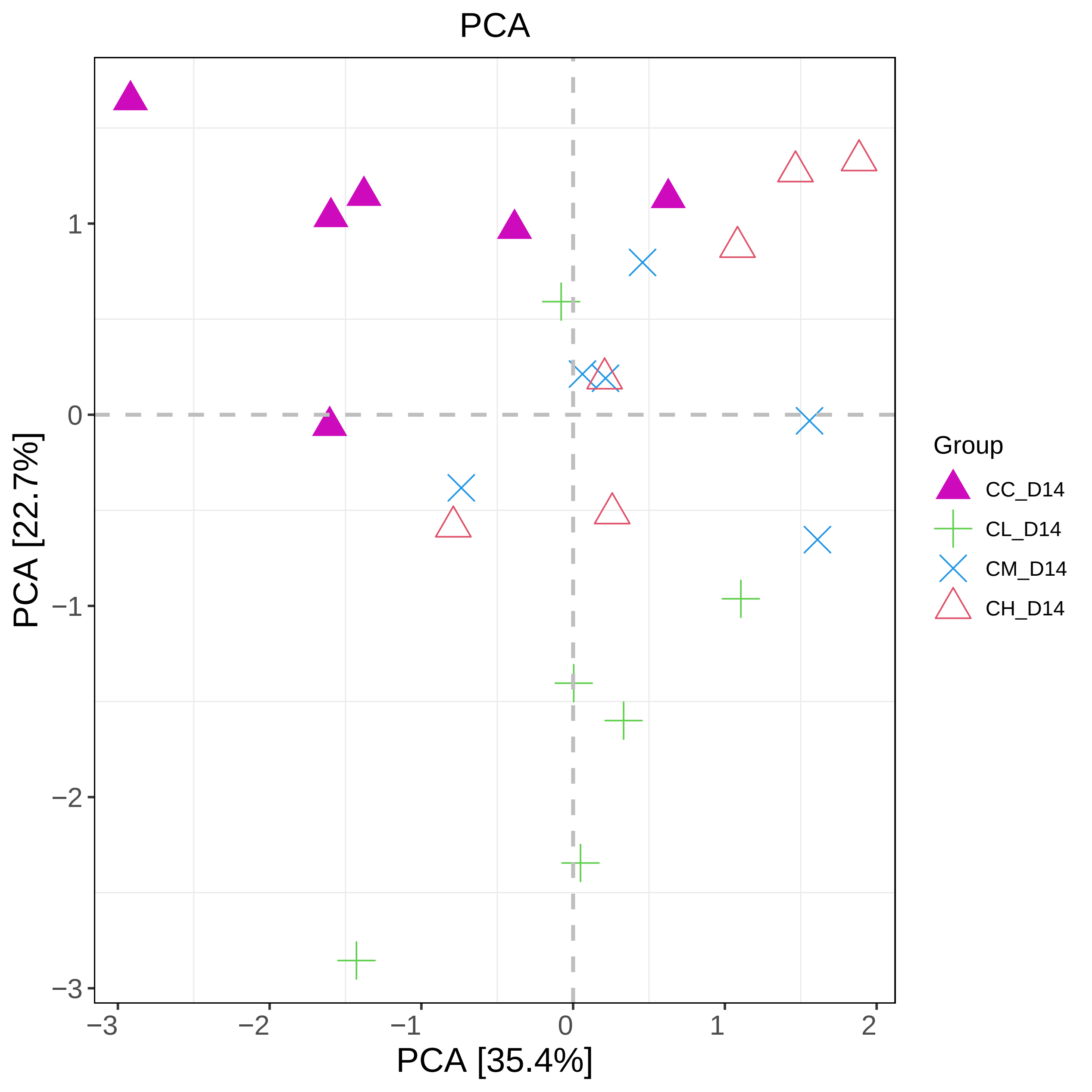
PCA
PCA [22.7%]
PCA [35.4%]
1
0
−1
−2
−3
−3
−2
−1
0
1
2
Group
CC_D14
CL_D14
CM_D14
CH_D14
