# Supplementary material for: Lycium barbarum-probiotic synergy alleviates chemotherapy-induced cancer-related fatigue via gut microbiota-metabolic axis regulation in mice
Source: Front Nutr. 2025 Jul 2;12:1613132. doi: 10.3389/fnut.2025.1613132 (PMC12263555; doi:10.3389/fnut.2025.1613132)
Supplement: Supplementary file 1 [file Supplementary_file_1.zip › Raw Data 1.ZIP!/Gut microbiota analysis data-1/罗宁生物扩增子测序报告/05.FunctionPrediction/FAPROTAX/Statistic/PCoA/FAPROTAX_PCoA_Jaccard_Group.pdf]

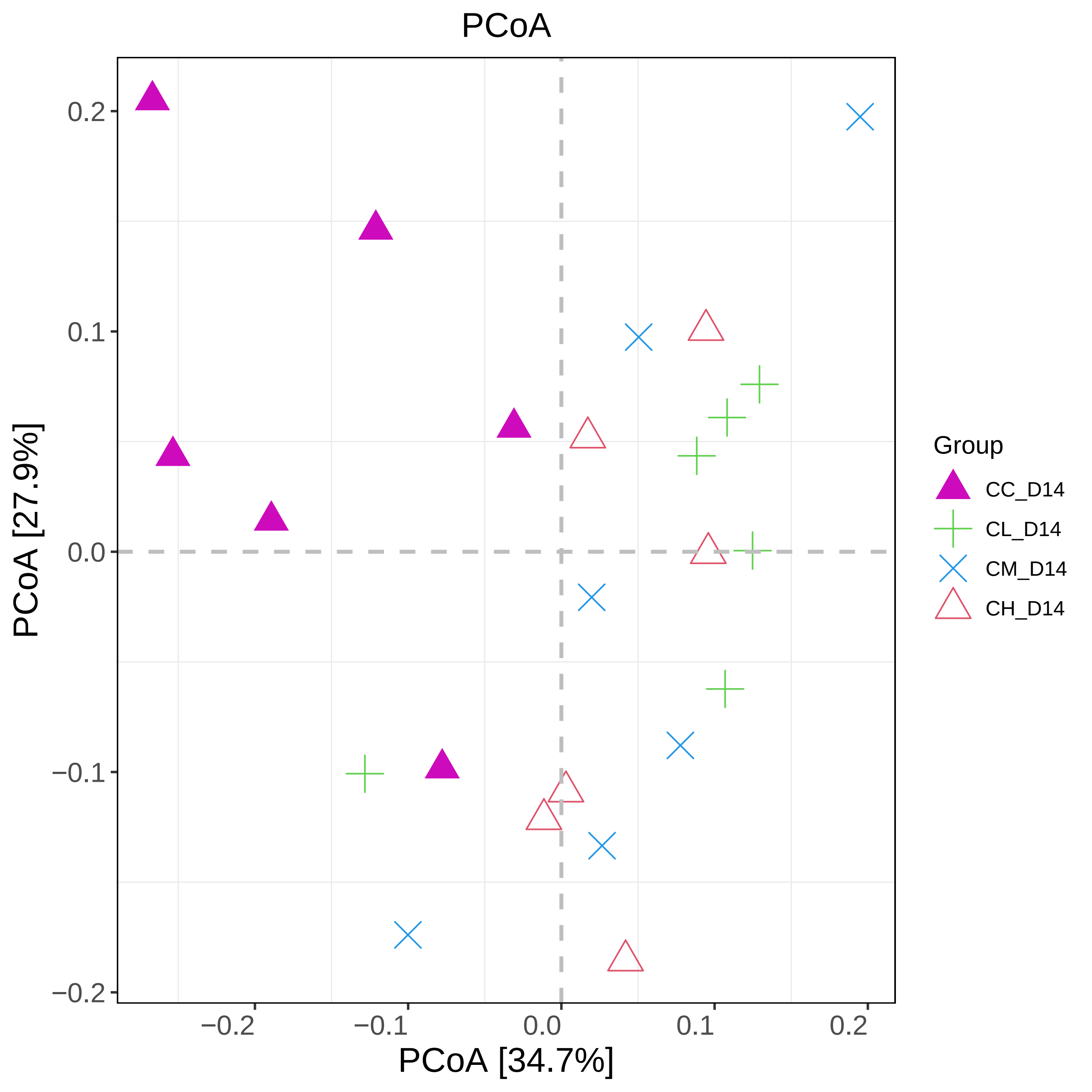
PCoA
PCoA [27.9%]
PCoA [34.7%]
0.2
0.1
0.0
−0.1
−0.2
−0.2
−0.1
0.0
0.1
0.2
Group
CC_D14
CL_D14
CM_D14
CH_D14
