# Supplementary material for: Lycium barbarum-probiotic synergy alleviates chemotherapy-induced cancer-related fatigue via gut microbiota-metabolic axis regulation in mice
Source: Front Nutr. 2025 Jul 2;12:1613132. doi: 10.3389/fnut.2025.1613132 (PMC12263555; doi:10.3389/fnut.2025.1613132)
Supplement: Supplementary file 1 [file Supplementary_file_1.zip › Raw Data 1.ZIP!/Gut microbiota analysis data-1/罗宁生物扩增子测序报告/05.FunctionPrediction/FAPROTAX/Statistic/RandomFroest/FAPROTAX_randomforest.pdf]

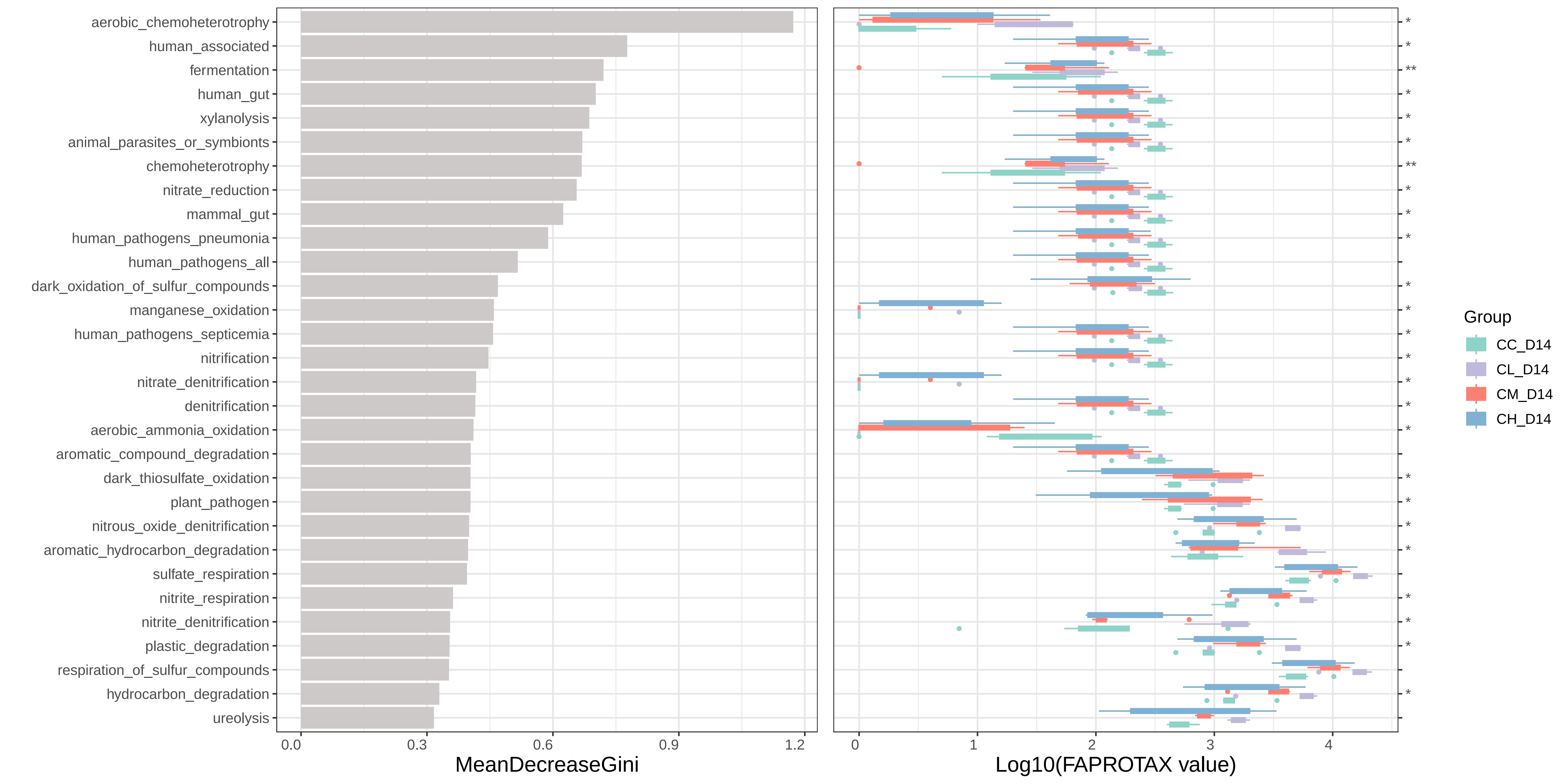

aerobic_chemoheterotrophy
human_associated
fermentation
human_gut
xylanolysis
animal_parasites_or_symbionts
chemoheterotrophy
nitrate_reduction
mammal_gut
human_pathogens_pneumonia
human_pathogens_all
dark_oxidation_of_sulfur_compounds
manganese_oxidation
human_pathogens_septicemia
nitrification
nitrate_denitrification
denitrification
aerobic_ammonia_oxidation
aromatic_compound_degradation
dark_thiosulfate_oxidation
plant_pathogen
nitrous_oxide_denitrification
aromatic_hydrocarbon_degradation
sulfate_respiration
nitrite_respiration
nitrite_denitrification
plastic_degradation
respiration_of_sulfur_compounds
hydrocarbon_degradation
ureolysis
0.0
0.3
0.6
0.9
1.2
MeanDecreaseGini
0
1
2
3
4
Log10(FAPROTAX value)
Group
CC_D14
CL_D14
CM_D14
CH_D14
