# Supplementary material for: Lycium barbarum-probiotic synergy alleviates chemotherapy-induced cancer-related fatigue via gut microbiota-metabolic axis regulation in mice
Source: Front Nutr. 2025 Jul 2;12:1613132. doi: 10.3389/fnut.2025.1613132 (PMC12263555; doi:10.3389/fnut.2025.1613132)
Supplement: Supplementary file 1 [file Supplementary_file_1.zip › Raw Data 1.ZIP!/Gut microbiota analysis data-1/罗宁生物扩增子测序报告/05.FunctionPrediction/FAPROTAX/Summary/FAPROTAX_BarPlot_Group.pdf]

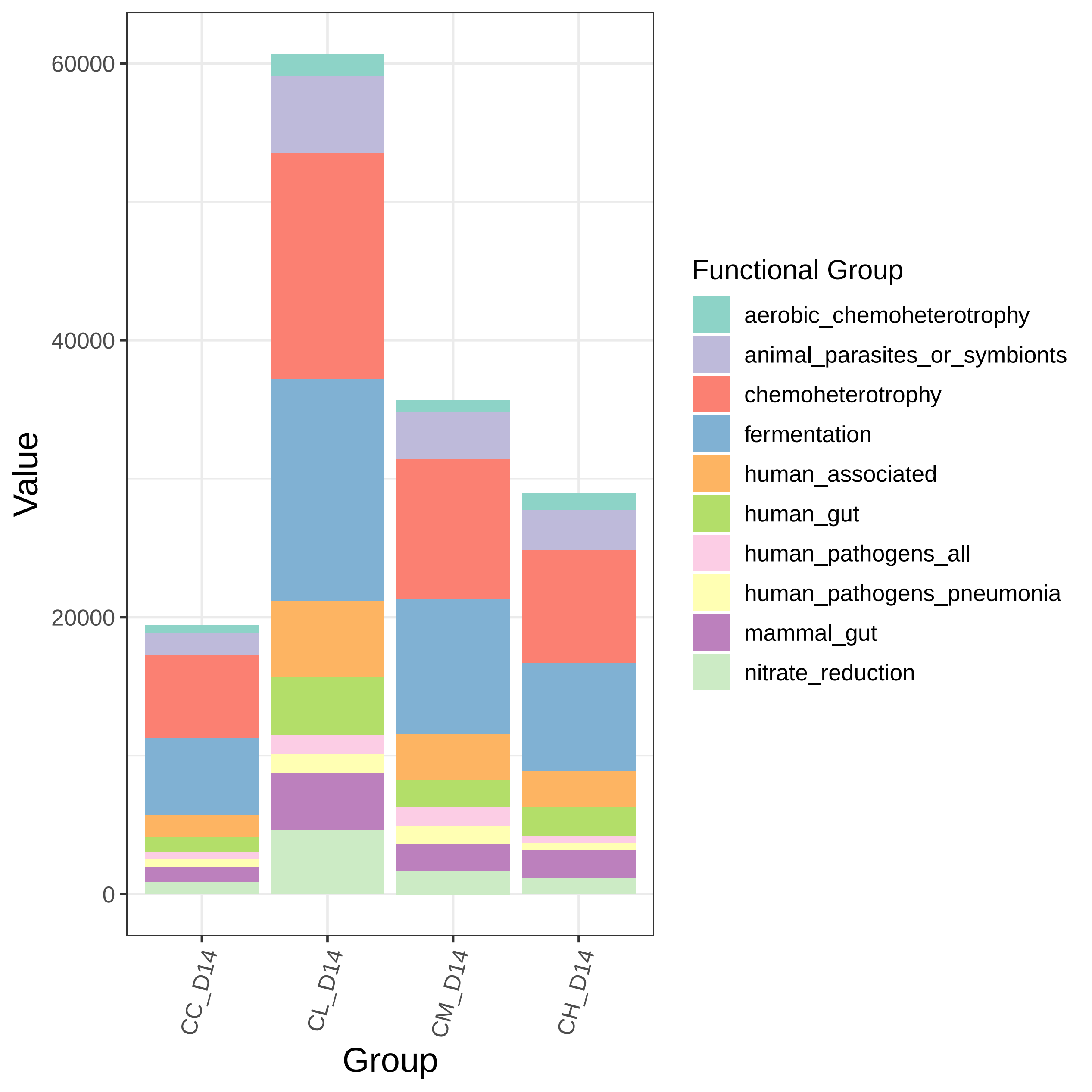
Value
60000
40000
20000
0
CC_D14
CL_D14
CM_D14
CH_D14
Group
Functional Group
aerobic_chemoheterotrophy
animal_parasites_or_symbionts
chemoheterotrophy
fermentation
human_associated
human_gut
human_pathogens_all
human_pathogens_pneumonia
mammal_gut
nitrate_reduction
