# Supplementary material for: Lycium barbarum-probiotic synergy alleviates chemotherapy-induced cancer-related fatigue via gut microbiota-metabolic axis regulation in mice
Source: Front Nutr. 2025 Jul 2;12:1613132. doi: 10.3389/fnut.2025.1613132 (PMC12263555; doi:10.3389/fnut.2025.1613132)
Supplement: Supplementary file 1 [file Supplementary_file_1.zip › Raw Data 1.ZIP!/Gut microbiota analysis data-1/罗宁生物扩增子测序报告/05.FunctionPrediction/PICRUSt2/Statistic/PCA/PICRUSt2_PCA.pdf]

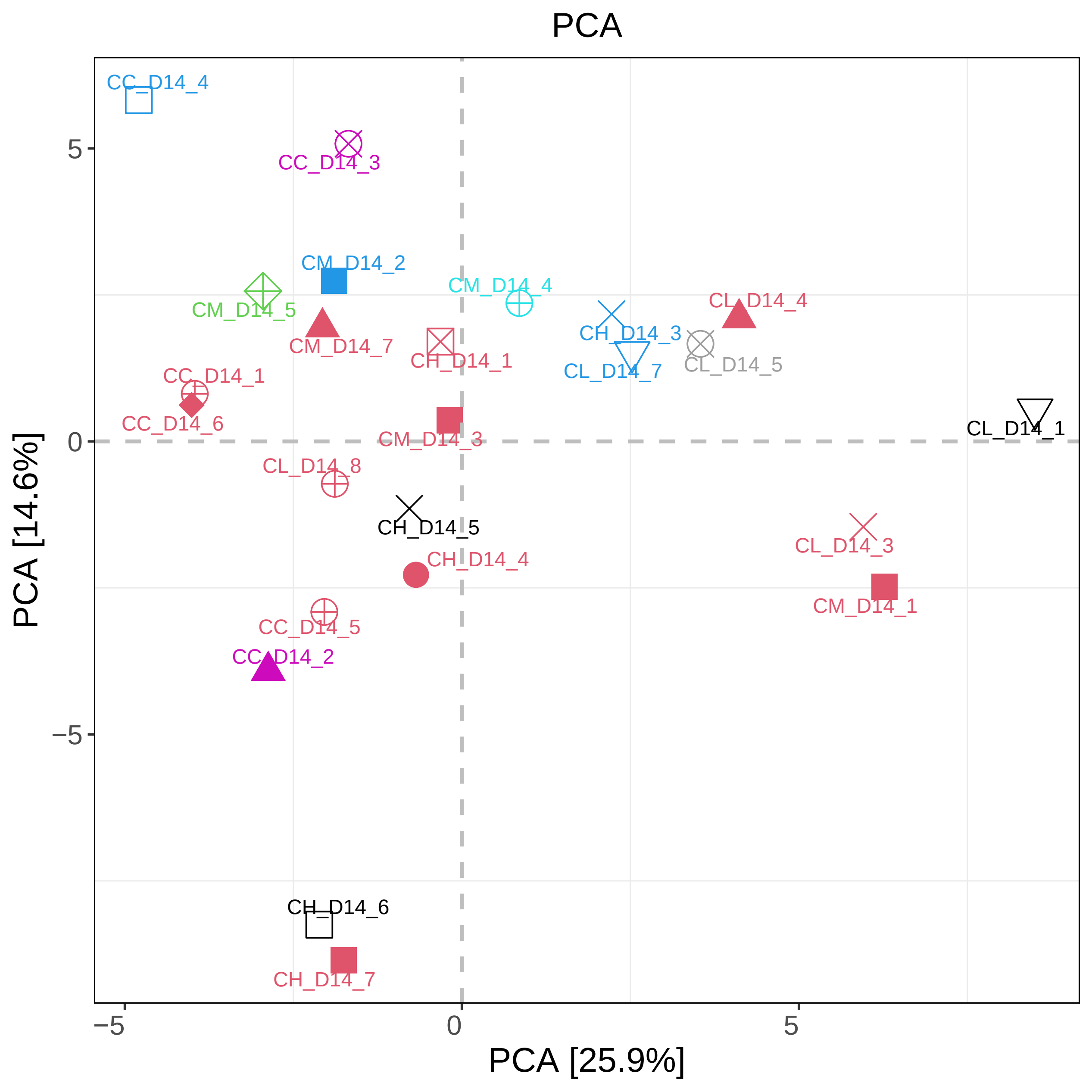
PCA
PCA [14.6%]
PCA [25.9%]
5
0
−5
−5
0
5
CC_D14_4
CC_D14_3
CM_D14_2
CM_D14_4
CM_D14_5
CM_D14_7
CL_D14_4
CH_D14_3
CH_D14_1
CL_D14_5
CL_D14_7
CC_D14_1
CC_D14_6
CM_D14_3
CL_D14_1
CL_D14_8
CH_D14_5
CL_D14_3
CH_D14_4
CM_D14_1
CC_D14_5
CC_D14_2
CH_D14_6
CH_D14_7
