# Supplementary material for: Lycium barbarum-probiotic synergy alleviates chemotherapy-induced cancer-related fatigue via gut microbiota-metabolic axis regulation in mice
Source: Front Nutr. 2025 Jul 2;12:1613132. doi: 10.3389/fnut.2025.1613132 (PMC12263555; doi:10.3389/fnut.2025.1613132)
Supplement: Supplementary file 1 [file Supplementary_file_1.zip › Raw Data 1.ZIP!/Gut microbiota analysis data-1/罗宁生物扩增子测序报告/05.FunctionPrediction/PICRUSt2/Statistic/RandomFroest/randomforest_KEGG.pdf]

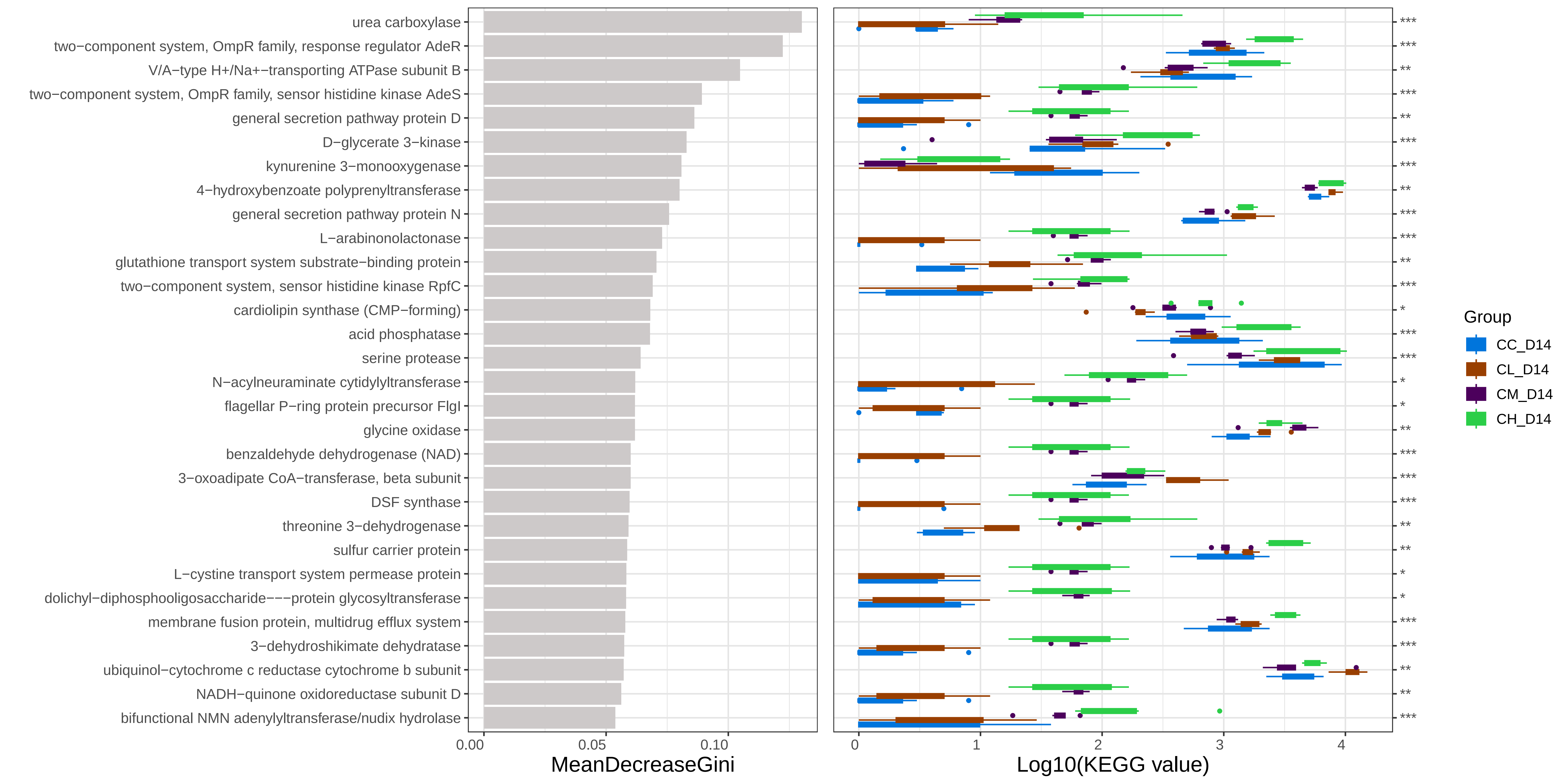

urea carboxylase
two−component system, OmpR family, response regulator AdeR
V/A−type H+/Na+−transporting ATPase subunit B
two−component system, OmpR family, sensor histidine kinase AdeS
general secretion pathway protein D
D−glycerate 3−kinase
kynurenine 3−monooxygenase
4−hydroxybenzoate polyprenyltransferase
general secretion pathway protein N
L−arabinonolactonase
glutathione transport system substrate−binding protein
two−component system, sensor histidine kinase RpfC
cardiolipin synthase (CMP−forming)
acid phosphatase
serine protease
N−acylneuraminate cytidylyltransferase
flagellar P−ring protein precursor FlgI
glycine oxidase
benzaldehyde dehydrogenase (NAD)
3−oxoadipate CoA−transferase, beta subunit
DSF synthase
threonine 3−dehydrogenase
sulfur carrier protein
L−cystine transport system permease protein
dolichyl−diphosphooligosaccharide−−−protein glycosyltransferase
membrane fusion protein, multidrug efflux system
3−dehydroshikimate dehydratase
ubiquinol−cytochrome c reductase cytochrome b subunit
NADH−quinone oxidoreductase subunit D
bifunctional NMN adenylyltransferase/nudix hydrolase
0.00
0.05
0.10
MeanDecreaseGini
0
1
2
3
4
Log10(KEGG value)
***
***
**
***
**
***
***
**
***
***
**
***
*
***
***
*
*
**
***
***
***
**
**
*
*
***
***
**
**
***
Group
CC_D14
CL_D14
CM_D14
CH_D14
