# Supplementary material for: Lycium barbarum-probiotic synergy alleviates chemotherapy-induced cancer-related fatigue via gut microbiota-metabolic axis regulation in mice
Source: Front Nutr. 2025 Jul 2;12:1613132. doi: 10.3389/fnut.2025.1613132 (PMC12263555; doi:10.3389/fnut.2025.1613132)
Supplement: Supplementary file 1 [file Supplementary_file_1.zip › Raw Data 1.ZIP!/Gut microbiota analysis data-1/罗宁生物扩增子测序报告/05.FunctionPrediction/PICRUSt2/Summary/KO/KO_Heatmap.pdf]

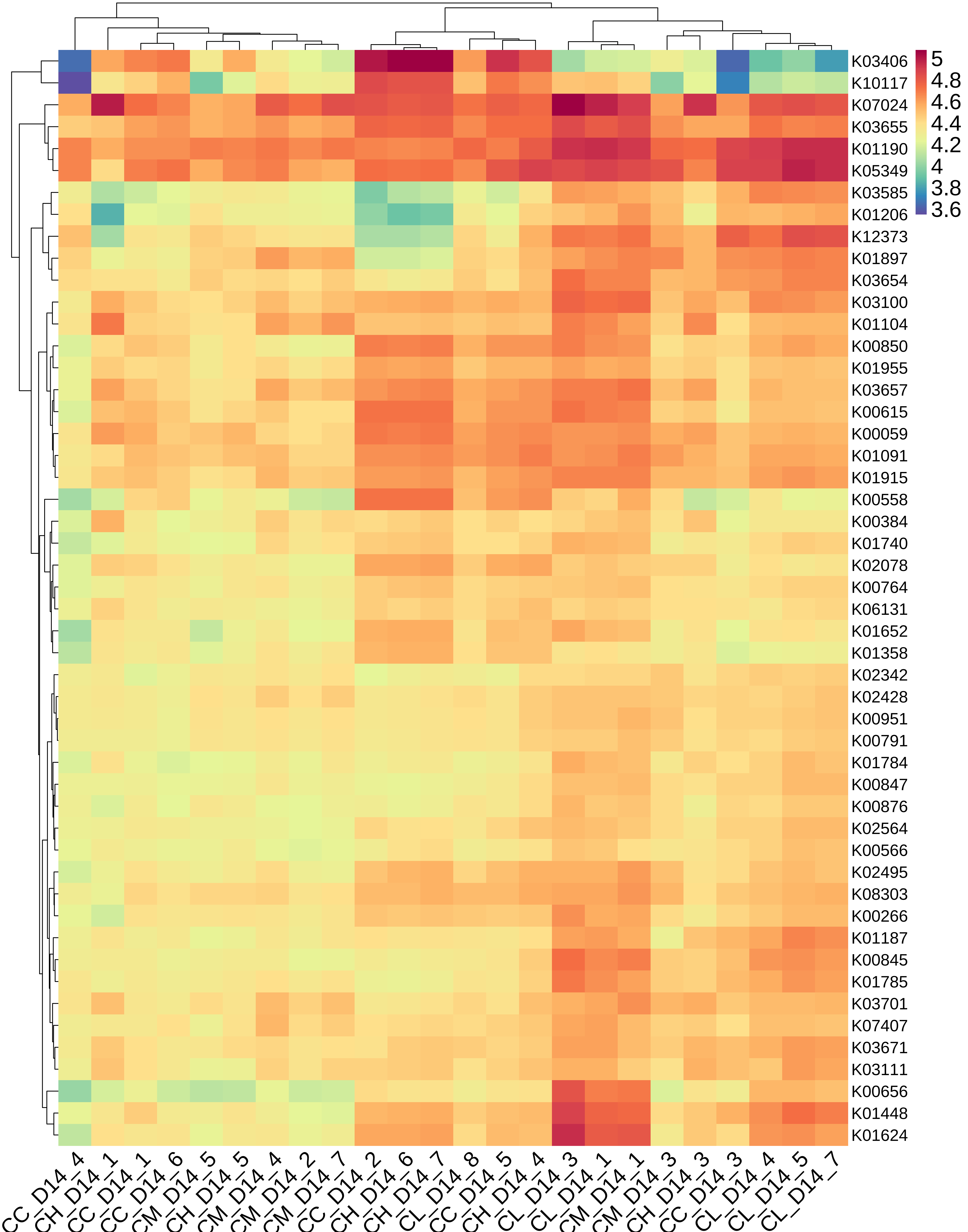
5
4.8
4.6
4.4
4.2
4
3.8
3.6
K03406
K10117
K07024
K03655
K01190
K05349
K03585
K01206
K12373
K01897
K03654
K03100
K01104
K00850
K01955
K03657
K00615
K00059
K01091
K01915
K00558
K00384
K01740
K02078
K00764
K06131
K01652
K01358
K02342
K02428
K00951
K00791
K01784
K00847
K00876
K02564
K00566
K02495
K08303
K00266
K01187
K00845
K01785
K03701
K07407
K03671
K03111
K00656
K01448
K01624
CC_D14_4
CH_D14_1
CC_D14_1
CC_D14_6
CM_D14_5
CH_D14_5
CM_D14_4
CM_D14_2
CM_D14_7
CC_D14_2
CH_D14_6
CH_D14_7
CL_D14_8
CC_D14_5
CH_D14_4
CL_D14_3
CL_D14_1
CM_D14_1
CM_D14_3
CH_D14_3
CC_D14_3
CL_D14_4
CL_D14_5
CL_D14_7
