# Supplementary material for: Lycium barbarum-probiotic synergy alleviates chemotherapy-induced cancer-related fatigue via gut microbiota-metabolic axis regulation in mice
Source: Front Nutr. 2025 Jul 2;12:1613132. doi: 10.3389/fnut.2025.1613132 (PMC12263555; doi:10.3389/fnut.2025.1613132)
Supplement: Supplementary file 1 [file Supplementary_file_1.zip › Raw Data 1.ZIP!/Gut microbiota analysis data-1/罗宁生物扩增子测序报告/05.FunctionPrediction/PICRUSt2/Summary/LEVEL_3/LEVEL_3.pdf]

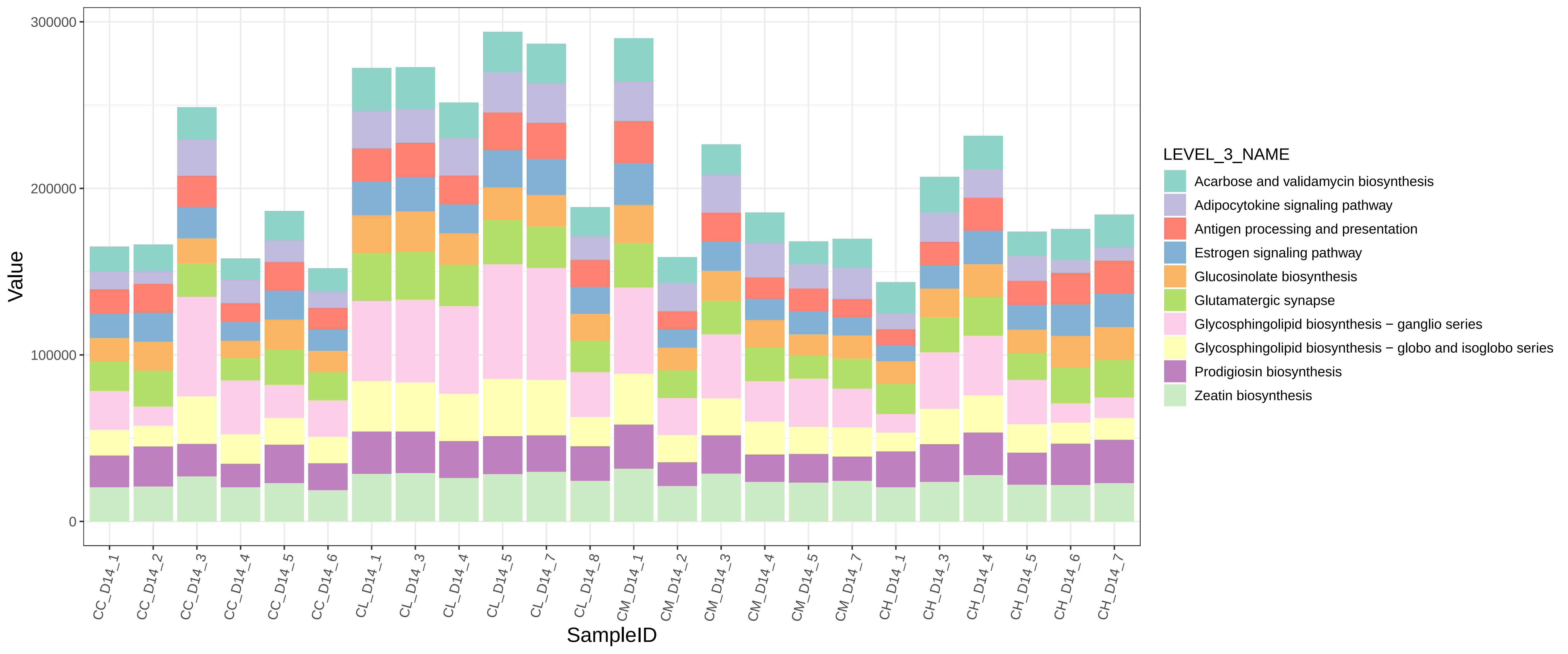

Value
300000
200000
100000
0
SampleID
CC_D14_1
CC_D14_2
CC_D14_3
CC_D14_4
CC_D14_5
CC_D14_6
CL_D14_1
CL_D14_3
CL_D14_4
CL_D14_5
CL_D14_7
CL_D14_8
CM_D14_1
CM_D14_2
CM_D14_3
CM_D14_4
CM_D14_5
CM_D14_7
CH_D14_1
CH_D14_3
CH_D14_4
CH_D14_5
CH_D14_6
CH_D14_7
LEVEL_3_NAME
Acarbose and validamycin biosynthesis
Adipocytokine signaling pathway
Antigen processing and presentation
Estrogen signaling pathway
Glucosinolate biosynthesis
Glutamatergic synapse
Glycosphingolipid biosynthesis − ganglio series
Glycosphingolipid biosynthesis − globo and isoglobo series
Prodigiosin biosynthesis
Zeatin biosynthesis
