# Supplementary material for: Lycium barbarum-probiotic synergy alleviates chemotherapy-induced cancer-related fatigue via gut microbiota-metabolic axis regulation in mice
Source: Front Nutr. 2025 Jul 2;12:1613132. doi: 10.3389/fnut.2025.1613132 (PMC12263555; doi:10.3389/fnut.2025.1613132)
Supplement: Supplementary file 1 [file Supplementary_file_1.zip › Raw Data 1.ZIP!/Gut microbiota analysis data-1/罗宁生物扩增子测序报告/05.FunctionPrediction/PICRUSt2/Summary/LEVEL_3/LEVEL_3_Group.pdf]

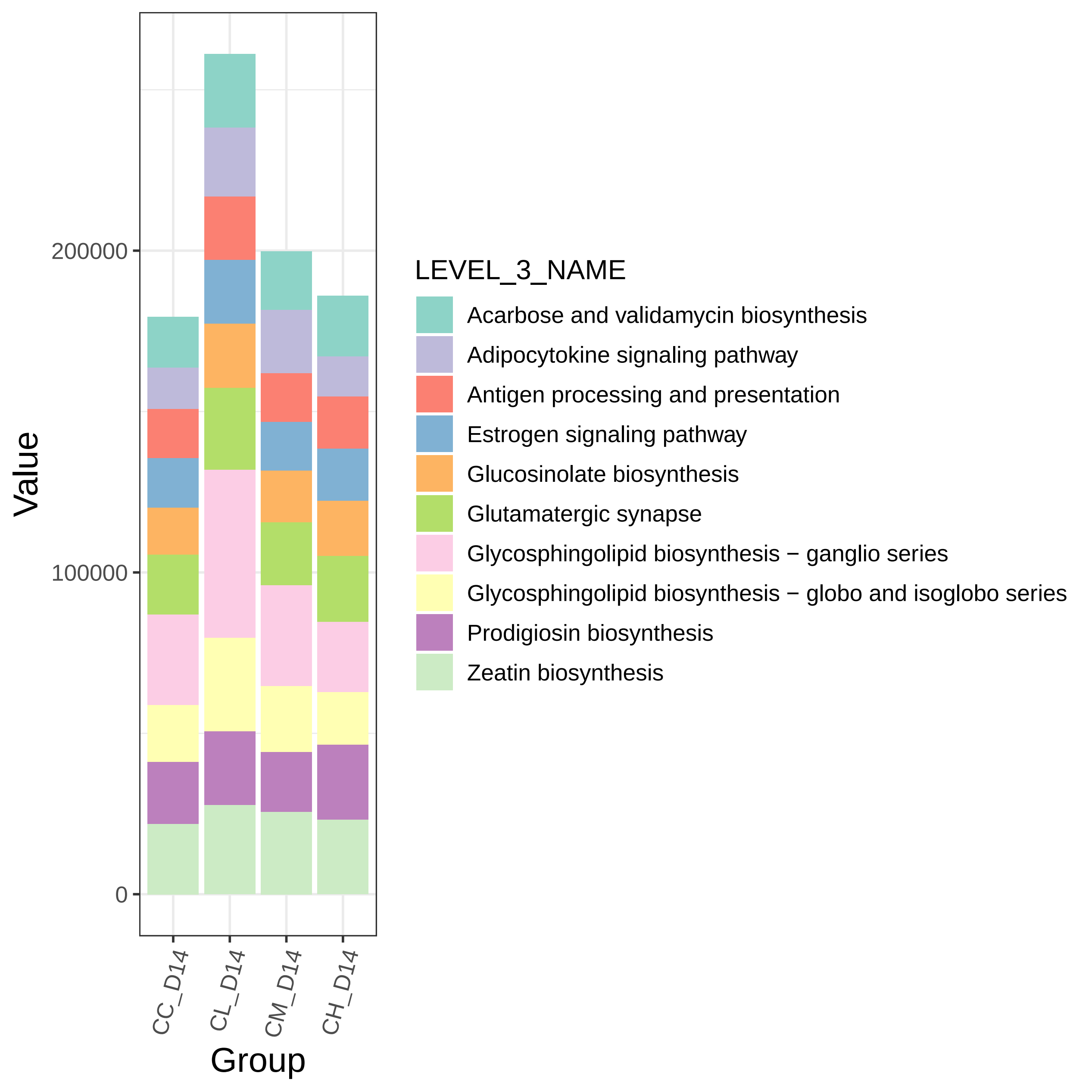

Value
200000
100000
0
CC_D14
CL_D14
CM_D14
CH_D14
Group
LEVEL_3_NAME
Acarbose and validamycin biosynthesis
Adipocytokine signaling pathway
Antigen processing and presentation
Estrogen signaling pathway
Glucosinolate biosynthesis
Glutamatergic synapse
Glycosphingolipid biosynthesis − ganglio series
Glycosphingolipid biosynthesis − globo and isoglobo series
Prodigiosin biosynthesis
Zeatin biosynthesis
